# Anopheline salivary protein genes and gene families: an evolutionary overview after the whole genome sequence of sixteen Anopheles species

**DOI:** 10.1186/s12864-017-3579-8

**Published:** 2017-02-13

**Authors:** Bruno Arcà, Fabrizio Lombardo, Claudio J. Struchiner, José M. C. Ribeiro

**Affiliations:** 1 Department of Public Health and Infectious Diseases – Division of Parasitology, Sapienza University, Piazzale Aldo Moro 5, 00185 Rome, Italy; 2 Fundação Oswaldo Cruz, Avenida Brasil, 4365 Rio de Janeiro, Brazil; 3 Instituto de Medicina Social, Universidade do Estado do Rio de Janeiro, Rio de Janeiro, Brazil; 4 Laboratory of Malaria and Vector Research, National Institute of Allergy and Infectious Diseases, 12735 Twinbrook Parkway, Rockville, MD 20852 USA

**Keywords:** Salivary glands, Salivary proteins, Anophelines, Mosquito saliva, Vector biology, Evolution, Salivary markers, Human exposure to malaria vectors, Positive selection

## Abstract

**Background:**

Mosquito saliva is a complex cocktail whose pharmacological properties play an essential role in blood feeding by counteracting host physiological response to tissue injury. Moreover, vector borne pathogens are transmitted to vertebrates and exposed to their immune system in the context of mosquito saliva which, in virtue of its immunomodulatory properties, can modify the local environment at the feeding site and eventually affect pathogen transmission. In addition, the host antibody response to salivary proteins may be used to assess human exposure to mosquito vectors. Even though the role of quite a few mosquito salivary proteins has been clarified in the last decade, we still completely ignore the physiological role of many of them as well as the extent of their involvement in the complex interactions taking place between the mosquito vectors, the pathogens they transmit and the vertebrate host. The recent release of the genomes of 16 Anopheles species offered the opportunity to get insights into function and evolution of salivary protein families in anopheline mosquitoes.

**Results:**

Orthologues of fifty three *Anopheles gambiae* salivary proteins were retrieved and annotated from 18 additional anopheline species belonging to the three subgenera *Cellia, Anopheles*, and *Nyssorhynchus*. Our analysis included 824 full-length salivary proteins from 24 different families and allowed the identification of 79 novel salivary genes and re-annotation of 379 wrong predictions. The comparative, structural and phylogenetic analyses yielded an unprecedented view of the anopheline salivary repertoires and of their evolution over 100 million years of anopheline radiation shedding light on mechanisms and evolutionary forces that contributed shaping the anopheline sialomes.

**Conclusions:**

We provide here a comprehensive description, classification and evolutionary overview of the main anopheline salivary protein families and identify two novel candidate markers of human exposure to malaria vectors worldwide. This anopheline sialome catalogue, which is easily accessible as hyperlinked spreadsheet, is expected to be useful to the vector biology community and to improve the capacity to gain a deeper understanding of mosquito salivary proteins facilitating their possible exploitation for epidemiological and/or pathogen-vector-host interaction studies.

**Electronic supplementary material:**

The online version of this article (doi:10.1186/s12864-017-3579-8) contains supplementary material, which is available to authorized users.

## Background

Anopheline mosquitoes are responsible for the transmission of human malaria, a disease which despite a significant decline in the last 15 years still caused over two hundred million new cases and around half a million deaths in 2015 [1]. The malaria parasite *Plasmodium* is ingested by the mosquito vector along with the blood meal while feeding on an infected individual. After gametogenesis and fertilization, taking place in the midgut lumen, the ookynetes traverse the monolayer of midgut cells and lodge below the basal lamina, where they differentiate into oocysts [2]. Mature oocysts release into the hemolymph thousands of sporozoites that specifically invade the mosquito salivary glands reaching the secretory cavity [3]. In order to get its next blood meal the mosquito penetrates the skin of a new host with the mouth parts and, while probing and feeding, salivates releasing sporozoites and transmitting the disease.

The saliva of blood feeding arthropods is a complex cocktail whose antihemostatic, antiinflammatory and immunomodulatory properties play a crucial role in counterbalancing the physiological host response to tissue injury and in facilitating successful accomplishment of blood feeding [4–6]. Moreover, pathogens are deposited into the skin and exposed to the vertebrate host immune system in the context of arthropod saliva. These vector salivary components can modify the feeding site and may affect the transmission of pathogens as diverse as arboviruses, bacteria and protozoan parasites [7–12], pointing out the possible exploitation of vector salivary proteins as potential vaccine targets [13–16]. Finally, inoculation of arthropod salivary proteins triggers in vertebrate hosts an antibody response which can be used as a biomarker of host exposure to vector bites and may represent a useful tool for epidemiological studies and evaluation of efficacy of vector control interventions [7, 17].

As far as anopheline mosquitoes are concerned the salivary protein repertoires (sialomes) of relevant malaria vectors as *Anopheles gambiae*, *An. funestus*, *An. stephensi* and *An. darlingi* have been previously characterized by classical transcriptome analyses based on Sanger sequencing [18–23] and by a few proteomic studies [24–28]. The anopheline for which a more comprehensive sialome information is available is certainly *An. gambiae* where PCR-based tissue-specific expression profiling and transcriptome analyses of salivary glands of both sexes [18, 20] allowed to distinguish: (i) genes specifically expressed or highly enriched in female salivary glands (FSG) and, therefore, most likely involved in blood feeding; (ii) genes expressed in both FSG and male salivary glands (MSG) and presumably involved in sugar digestion, in containing microbial growth or in other more general organ-specific physiological functions. The *An. gambiae* sialome presently includes over 70 secreted proteins, a number that may be susceptible to increase using up-to-date next generation sequencing techniques, as suggested by previous studies on the culicine mosquito *Aedes aegypti* [29, 30]. Surprisingly, although the role of quite a few anopheline salivary proteins has been clarified [22] we still have no insights into the functions of approximately forty per cent of them.

The recent release of the genomes of 16 anopheles species [31] offered the unique opportunity to get insights into function and evolution of salivary genes and salivary protein families in anopheline mosquitoes. Based on the above mentioned transcriptomic and gene expression studies on the African malaria mosquito *An. gambiae* we selected 53 salivary proteins, whose expression is specific or highly enriched in the mosquito salivary glands, and identified/annotated orthologues from 18 additional anopheline species, which include malaria vectors from different geographic areas as well as two African non-vector species (*An. quadriannulatus* and *An. christyi*). All three Anopheles subgenera, that is *Cellia* (series Pyrethophorus, Myzomyia, Neocellia, Neomyzomyia), *Anopheles* and *Nyssorhynchus* (with the New World species *An. albimanus* and *An. darlingi*) are represented, providing the opportunity to look at the evolution of salivary genes in the time frame of anopheline radiation, which is estimated to have started approximately 100 million years ago. We used this information for sequence comparisons, phylogenetic analyses and for secondary structure prediction (when relevant) to evaluate divergence, evolution and gene gain/loss events which took place during anopheline radiation. We report here the results of our analysis, which provides detailed information and consistent classification on anopheline salivary proteins belonging to at least 24 different families. We are confident that this anopheline sialome catalogue will be useful to the vector biology community and it is expected to improve the capacity to gain a more accurate and deeper understanding of mosquito salivary proteins and to facilitate their possible exploitation for epidemiological and/or pathogen-vector-host interaction studies.

## Results and discussion

**Fig. 1:**
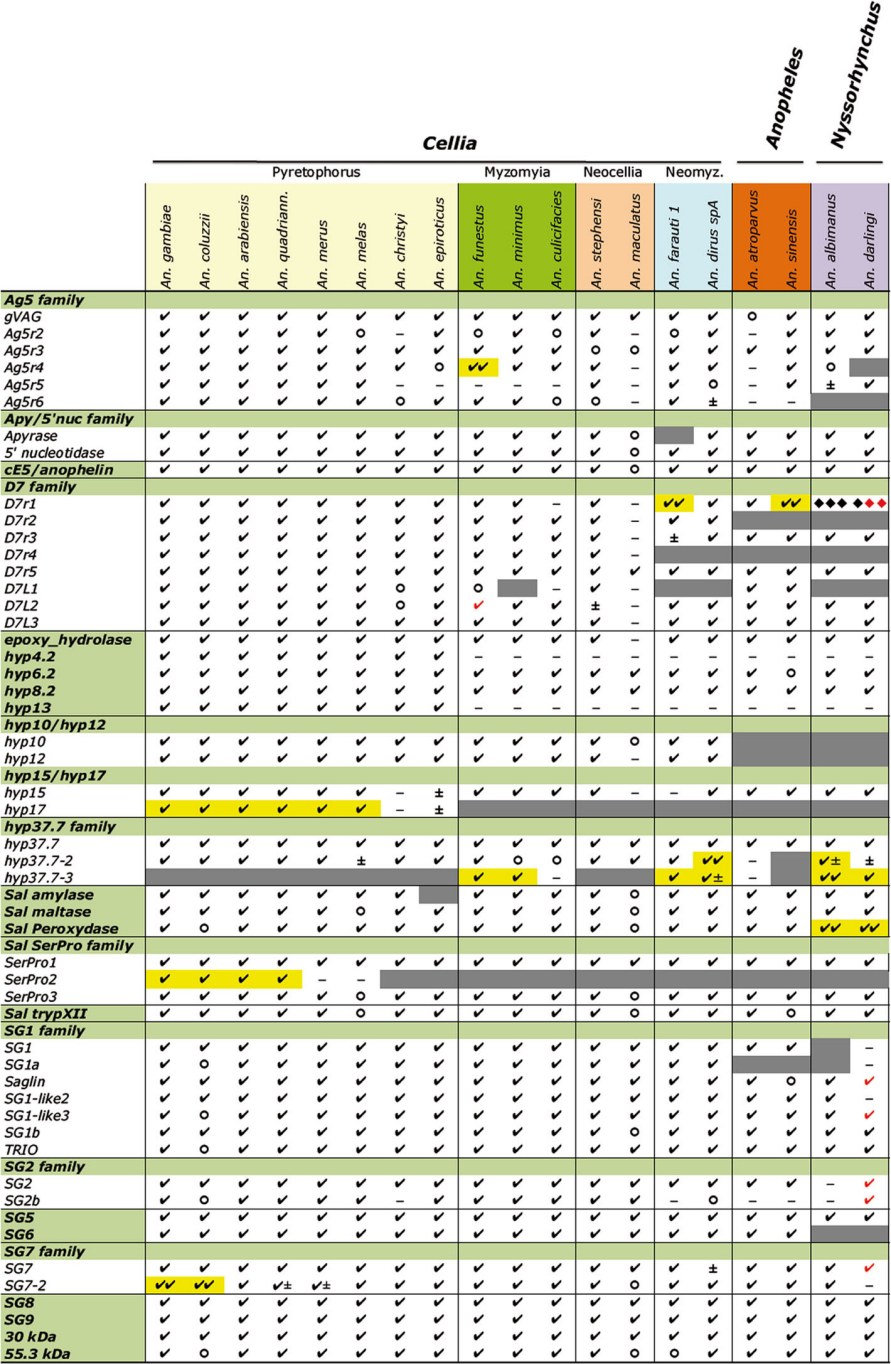
Distribution of the *An. gambiae* salivary proteins orthologues in anophelines. The selected *An. gambiae* proteins (*left*) were used to search orthologues in the genomes of the different anopheline species (*top*). Retrieval of full-length (✓), partial (○) or frameshift containing (±) coding sequences is reported. *Red* denotes genes not found but available from previous transcriptomes [19, 21]. Gene absence/loss and gene gain/duplication are highlighted in *grey* and *yellow*, respectively. Genes that were not found or not assessable (i.e. for which there was no clear evidence of gene loss) are indicated with a minus (−). Diamonds (♦) were used to indicate the shorter D7r typical of *Nyssorhynchus* species

Based on the previously assembled *An. gambiae* salivary gene catalogue [18], and excluding low complexity genes (e.g. salivary mucins), we selected 53 *An. gambiae* salivary proteins (Additional file 1) and searched the genomes of the eighteen anopheline species listed in the methods section using tblastn at the VectorBase web site [32]. Whenever possible, orthologous genes were retrieved through the genome browser and manually annotated using the Artemis tool [33]. The results of searches and annotation are summarized in Fig. 1, which shows for each species if the coding region: (i) could be identified (either as full-length, partial or with frameshifts); (ii) was not assessable (i.e. could not be identified but, at the same time, there was no convincing indication of gene loss, for example because of small genes and possible high divergence or due to highly fragmented or incomplete genome assembly); (iii) was absent (gene loss, as also evaluated from flanking genes); (iv) was duplicated (gene gain). Our analysis, which includes a total of 824 salivary proteins belonging to at least 24 different protein families from 19 anopheline mosquito species, allowed to re-annotate 379 wrongly predicted transcripts and to identify 79 novel salivary protein-coding genes not previously annotated in VectorBase (transcripts v1.00, June-October 2015 depending on the species). The coding sequences of the 824 full-length proteins were used to create an hyperlinked excel spreadsheet which carries sequences, accession numbers and several additional useful information (Additional file 2).

The proteins were classified in four main categories: (1) Enzymes, (2) Widespread in blood feeding arthropods, (3) Conserved mosquito families (i.e. only found in mosquitoes) and (4) Anopheline-specific families (i.e. restricted to anopheline species). In the following paragraphs we will describe the main anopheline salivary protein families following this classification and will be referring often to Fig. 1 and Additional file 2. However, it is first useful clarifying that during the initial sialotranscriptome studies on *An. gambiae* [34–36] the suffix gSG (gambiae Salivary Gene) followed by a number was used to identify *An. gambiae* salivary gland genes/proteins with no similarity to known genes/proteins in databases. This has sometimes generated confusion in the following literature. Here we will use gSGX to refer to the *An. gambiae* gene/protein X and SGX to generically indicate the anopheline gene/protein family X; both the family name and species name will be used to identify a family member from a species different from *An. gambiae*. Moreover, in the following discussion on salivary proteins and protein families we will often report the range of percent identity among anophelines: in all cases the comparisons between species of the *An. gambiae* complex (*An. gambiae s.s.*, *An. coluzzii*, *An. arabiensis*, *An. quadriannulatus*, *An. merus* and *An. melas*), which are very close to each other, were not considered.

### Enzymes

#### Apyrase and 5′-nucleotidase

Apyrases (ATP diphosphohydrolases) are enzymes that catalyze the hydrolysis of ATP and ADP to AMP and inorganic phosphate. ATP and ADP, released in the extracellular environment by broken cells following tissue injury, play an important role as triggers of hemostasis and inflammation. ATP acts as pain mediator and activates neutrophils, which aggregate and degranulate at the site of injury, whereas ADP is a powerful inducer of platelet aggregation. Moreover, ATP and ADP are further released by degranulating neutrophils and platelets. Perhaps for these reasons a salivary apyrase activity has been found in every group of hematophagous arthropods analyzed so far [6, 22, 37, 38], except the bovine feeding *Stomoxys calcitrans* [39] and *Haematobia irritans* [40]. As a good example of convergent evolution at least three different classes of apyrases are found in blood feeding arthropods: the CD39 class of fleas [41], the Cimex-type of bed bugs and sand flies [42, 43] and the apyrases of the 5′-nucleotidase family, first found in the mosquito *Ae. aegypti* [44] and then identified in other mosquitoes as well as in black flies, tsetse flies, triatomine bugs and ticks [22, 37, 38].

5′-nucleotidases (5′-ribonucleotide phosphohydrolases) are ubiquitous enzymes that hydrolyze 5′-nucleotides to nucleosides. They are usually GPI anchored through the C-terminus and play an important role in nucleotide metabolism converting extracellular nucleotides to corresponding nucleosides, which can easily traverse cell membranes. Therefore, as first shown for *Ae. aegypti* [44] and then confirmed for *An. gambiae* [45] mosquito apyrases evolved from a member of the 5′-nucleotidase family by gene duplication, loss of the C-terminus involved in GPI anchoring and acquisition of tissue- and sex-specific expression: this way a membrane-bound molecule originally involved in general nucleotide metabolism evolved to a secreted salivary protein playing crucial roles in blood feeding.

Mosquitoes, as first shown in *An. gambiae*, carry in their saliva two secreted members of the 5′nucleotidase family [34, 45]. The specific enzymatic activity of these two different *An. gambiae* proteins has never been experimentally verified. However, according to both PCR- and microarray-based expression studies, AGAP011971 was found specifically expressed in adult FSG whereas AGAP011026, although enriched in female glands, showed a more promiscuous expression pattern [35, 46]. For this reason the former has been considered and named as the apyrase and the latter as the 5′-nucleotidase of this mosquito. The two enzymes could act sequentially in anophelines as first suggested for the sandfly *Lutzomyia longipalpis* [47]. In this scenario the apyrase would hydrolyze the ATP/ADP released from injured tissues to AMP, and the 5′-nucleotidase might further convert the AMP to adenosine, which is not only an antagonist of platelet recruitment, adhesion and aggregation but also a potent vasoactive agent [47, 48].

Full-length orthologues of the *An. gambiae* salivary apyrase and 5′-nucleotidase were found in all the anopheline species analyzed here with only two exceptions: *An. maculatus,* where only truncated sequences could be identified, and *An. farauti*, where the apyrase gene was apparently lost, as indicated by careful inspection of the genomic region and the flanking genes (Fig. 1). This observation is somehow surprising considering the widespread occurrence and the key role of salivary apyrases in blood sucking arthropods. It may be interesting to verify if an apyrase activity is present in the saliva of *An. farauti*, i.e. if after the gene loss some other gene was recruited to hydrolyze ATP and ADP to AMP. Sequence comparisons showed a minimal amino acid identity of 63% among anopheline apyrases and 67% among 5′-nucleotidases, whereas the identity between apyrases and 5′-nucleotidases is in the range of 46 to 50%. Phylogenetic analysis of the aligned mature proteins showed two independent clades for apyrases and 5′-nucleotidases with strongly supported subclades for *Cellia, Anopheles* and *Nyssorhynchus* species (Additional file 3).

#### Epoxy hydrolase

Epoxy hydrolases (also known as epoxy hydratates) are enzymes that metabolize compounds containing an epoxide (i.e. a cyclic ether with a three atom ring) giving the corresponding dihydroxy through the addition of water. A transcript encoding an epoxy hydrolase (AGAP011970) was found during a sialotranscriptome analysis in *An. gambiae* and full-length orthologues were identified in all anophelines analyzed here but *An. maculatus* (Fig. 1, Additional file 2). Multiple alignment showed an overall conserved structure with amino acid identity in the range of 48–87%. Among these eighteen hypoxy hydrolases only six seem to contain a canonical secretory signal according to prediction analysis and, therefore, their secretion in the saliva of anopheline mosquitoes is likely but not certain. However, we included this enzyme in our salivary list for at least two reasons. First, the *An. gambiae* epoxy hydrolase AGAP011970 is located in close proximity and in reverse orientation to the salivary apyrase (the two starting Met are separated by only 354 bp) and the two genes show a very similar expression profile, both being specific or highly enriched in adult FSG [18, 46]. Second, epoxy hydrolases play relevant roles in the metabolism of epoxy-fatty acids, which are known to be involved in inflammation, hemostasis and pain [49].

#### Salivary amylase and maltase

Transcripts encoding amylase and maltase were found in *An. gambiae* and shown to be over-expressed in adult salivary glands of both sexes [18, 46]. Their function is most likely associated with sugar feeding and they may help digestion in the mosquito crop and midgut. Members of the same families are expressed in the salivary glands of the culicine *Ae. aegypti* [50, 51] and were found in all blood feeding Nematocera sialomes done so far [22]. Orthologues of the *An. gambiae* salivary amylase and maltase were found, either as full-length or partial, in all anophelines (Fig. 1, Additional file 2) with the only notable exception of *An. epiroticus*, where the salivary amylase was lost. It is possible that in this species another member of the family, placed in a different genomic context, was recruited to salivary expression and sugar feeding function after the loss of the ancestral gene. Overall salivary maltase proteins show well conserved size in anophelines (593–598 aa) with 78% minimal identity. On the contrary salivary amylases showed a lower identity (range 51–77%) and are rather heterogeneous in size: 871–880 aa within the *An. gambiae* complex, 598–628 aa in the two Neomyzomyia species *An. farauti* and *An. dirus*, and 724–789 aa in the other anophelines. The divergence and fast evolution of these genes suggests they may be under host immune pressure if secreted by female anophelines while probing, as is the case with *Ae. aegypti* [52].

#### Salivary peroxidase

The first member of the family was identified in the mosquito *An. albimanus* by classical biochemical purification followed by cDNA cloning [53, 54]. It was shown to be a heme peroxidase with catechol oxidase/peroxidase activity acting as a vasodilator by inactivating vasoconstriction agents such as noradrenaline and serotonin. Transcripts coding for enzymes of the same family, and supposedly having the same function, were then found through sialotranscriptome studies in *An. darlingi* [55] and *An. gambiae* [18]. The *An. gambiae* salivary peroxidase (AGAP010735) appeared specifically expressed in adult FSG [18, 46]. Several members of the heme peroxidase family are found in the anopheline genomes, nevertheless full-length orthologues were identified with good confidence in all species with the only exceptions of *An. coluzzii* and *An. maculatus* where only partial sequences could be reconstructed (Fig. 1). In *An. gambiae* two additional peroxidase genes were found in the same genomic region coding for AGAP010735, approximately 9 kB upstream and 19 kB downstream. For the first no information on the expression profile is available, whereas the second (AGAP010734) was found expressed in several tissues and significantly upregulated in Malpighian tubes [46]. For this reason these two additional *An. gambiae* peroxidase family members will not be considered here. A similar situation was found in the other anophelines with the exception of the two species of the subgenus *Nyssorhynchus* which carry a cluster of five peroxidase genes in a region of ~15 kb. We named the *An. albimanus* gene encoding the salivary peroxidase biochemically characterized by Ribeiro & Valenzuela [54] as anoalb_Sal_Perox (Additional file 2). However, the true orthologue of AGAP010735 seems to be a close gene that shows the highest identity to the *An. gambiae* salivary peroxidase (75% identity, 85% similarity) and was named anoalb_Sal_Perox2 (Additional file 4) to indicate that this mosquito may express two salivary peroxidases. The possibility that also other members of the cluster may have salivary expression cannot be ruled out, however, in the absence of additional information we did not consider the other three peroxidases as salivary and indicated them as Perox3, Perox4 and Perox5 in Additional file 4. The other New World species *An. darlingi* also carries a cluster of multiple genes, although only one peroxidase transcript was found in a previous sialotranscriptome study [21]. We hypothesize here that *An. albimanus* and *An. darlingi* may express two salivary peroxidases and tentatively classify this as a gene gain in Fig. 1. Future sialome studies on these two New World species, preferably employing next generation sequencing techniques, should help clarify whether this hypothesis is correct.

#### Salivary serine proteases

Four secreted trypsin-like serine proteases with highly enriched or specific expression in salivary glands and named Sal_SerPro1-3 and Sal_trypXII were found in *An. gambiae*. Serine proteases are found expressed in the salivary glands of mosquitoes and other blood feeding Nematocera [22] but their function is presently unknown since no members of this family has been biochemically characterized so far. Their function may be related to immunity, for example as prophenoloxidase activators, or to blood feeding, by interfering with the inflammatory pathways or affecting hemostasis as in the case of the anticoagulant serine protease tabserin from the horsefly *Tabanus yao* [56].

*Sal_SerPro1* (AGAP011912) and *Sal_SerPro2* (AGAP011914) were identified in *An. gambiae by* previous transcriptomes and are located on 3 L:44C, being separated by a 4.2 kb intergenic region. Careful examination of the genomic locus showed that between these two genes there is a third member of the family (AGAP011913) not previously identified in sialome analyses. All three genes show identical expression pattern with significant upregulation in both FSG and MSG [46] and for this reason we included also this third gene in our analysis and named it *Sal_SerPro3*. The expression in salivary glands of both sexes suggests a more likely involvement in innate immunity rather than in blood feeding. A similar expression pattern in both FSG and MSG was also found during a recent RNAseq analysis for the *Ae. aegypti* salivary serine proteases [30]. All three Sal_SerPro proteins also contain an amino-terminal CUB domain (Pfam: PF00431), a module of approximately 110 amino acids with four conserved cysteine residues that can be involved in oligomerization and/or recognition of substrates and binding partners. CUB domain-containing serine proteases have also been identified in a sialotranscriptome analysis of the mosquito *Ae. aegypti* and the presence of the CUB domain interpreted as possibly involved in specialized substrate recognition [29]. *Sal_SerPro1* and *Sal_SerPro3* orthologues were found in all eighteen anopheline species analyzed here. On the contrary, *Sal_SerPro2* was only found in some members of the *An. gambiae* species complex, but it was absent in all other anophelines (it should be noted that in *An. merus* and *An. melas* a third salivary serine protease was not found, most likely just because of the short contigs carrying the cluster) (Fig. 1). The *An. gambiae* Sal_SerPro2 is 49% identical to Sal_SerPro1 and 88% identical to Sal_SerPro3. Therefore, it it is likely that *Sal_SerPro1* and *Sal_SerPro3* were already present in the ancestral lineage of anophelines, which may have appeared >100 Mya before the complete splitting of Pangaea in Gondwana and Laurasia [57], whereas *Sal_SerPro2* originated “recently” by gene duplication of *Sal_SerPro3* in the progenitor of the *An. gambiae* species complex, i.e. around 2 Mya [58]. Multiple alignment of the forty Sal_SerPro polypeptides showed a well conserved overall structure (minimal identity 43.7%) and full conservation of the 16 cysteines and of the catalytic triad typical of serine proteases and consisting of histidine, aspartate and serine (H199, D249 and S341 in Sal_SerPro1, Additional file 5A). Phylogenetic analysis yielded two well supported independent clades, one including all anopheline Sal_SerPro1 and the other including all Sal_SerPro3 plus the Sal_SerPro2 from species of the *An. gambiae* complex (Additional file 5B).

A fourth FSG-specific trypsin-like serine protease was first identified in *An. gambiae* and named Sal_trypXII because of some similarity with Factor XII. Differently from the Sal_SerPro proteins it does not contain any additional conserved domain and, as previously reported, seems to undergo to a tissue- and sex-specific splicing that may play a role in tissue translation selectivity [18]. Orthologues, mostly full-length, were identified in all eighteen additional anophelines analyzed here (Fig. 1) and they share a minimal amino acid identity of 63%. Multiple alignment showed the presence and full conservation of the tripeptide motif (K/R)GD known for the ability to bind integrins. As other functional RGD motifs it is flanked by disulphide bonds able to form a peptide hairpin with the G at the apex [59]. If functional, it may affect platelet aggregation; however, the motif is immediately followed by the Serine that is part of the catalytic triad and, therefore, it may be buried and not available for the interaction (Additional file 6). Serine proteases containing RGD motifs are rather unusual, nevertheless it should be noted that thrombin is known to contain a buried RGD and has been suggested to be able to bind integrins through partial unfolding or after proteolitic digestion, which would expose the RGD to the solvent [60].

### Widespread among blood feeding arthropods

This group includes at least three protein families, namely Antigen 5 (Ag5), D7 and 30 kDa. The *D7* and *Ag5* are multigene families organized in clusters and originated by multiple gene duplication and divergent evolution. In *An. gambiae* there are eight *D7* and several *Ag5* family members, of which six will be considered here. Identification of orthologues in the different anopheline species was sometime tricky for these multigene families. We were mainly based on the gene order in the clusters and sequence comparisons to solve ambiguous cases of gene gain/loss and for classification and naming (Fig. 1, Additional file 2).

#### Antigen 5 family

The first mosquito salivary member of the *Ag5* family was identified in *An. gambiae* [35] and named *gVAG* (***g*** *ambiae* Venom AllerGen) because of its similarity to allergens from the venom of ants and wasps [61]. Since then transcripts encoding proteins of the family were found in sialotranscriptomes of most or all blood feeding arthropods analyzed so far [6, 22]. Ag5 proteins are part of the widely spread CAP superfamily, which includes Cysteine-rich secretory proteins from mammals, Antigen 5 from insects, and Pathogenesis-related proteins from plants and whose functions are highly diversified [62, 63]. Members of this family from the venoms of snakes, lizards and Conus snails have been shown to function as toxins, ion channels inhibitors or proteases [64, 65], and Ag5 proteins in the venoms of ants and wasps are powerful allergens to mammals [66, 67]. Only more recently the function of a few Ag5 family members from the saliva of blood feeding insects has been clarified. This is the case of the RGD-containing platelet inhibitors tabinhibitins from *Tabanus yao* [56], of a 27 kDa immunoglobulin-binding protein from *Stomoxys calcitrans* [68] (which may function as an inhibitor of the classic pathway of complement) and of Ag5 proteins from the saliva of Triatomines, that are copper-dependent antioxidant enzymes inhibiting neutrophil oxidative burst and collagen-induced platelet aggregation [69]. No salivary Ag5 family members from mosquitoes have been functionally characterized so far.

Insect genomes encode a quite large number of rather divergent *Ag5* family members and at least 19 are found in the genome of *An. gambiae*. Four *Ag5* members were previously found expressed in the *An. gambiae* salivary glands: *gVAG* (AGAP006421), *Ag5r2* (AGAP006419), *Ag5r4* (AGAP006420) and *Ag5r3* (AGAP003354). The first three form a cluster on 2 L-24D and are upregulated in both MSG and FSG; the fourth is located on 2R-15A, has lower level of expression in salivary glands and it is transcribed at similar level also in other tissues [18, 46]. Careful genome examination allowed to identify two additional family members, apparently the products of gene duplications of *Ag5r2* and *Ag5r3*: (i) AGAP006418 which is close to *Ag5r2* (~1.7 kb upstream), has similar expression pattern and high degree of amino acid identity (79%); (ii) AGAP013192 located ~3.2 kb downstream of *Ag5r3* and sharing with it identical expression pattern and high identity (85%). For these reasons, although AGAP006418 and AGAP013192 were not described earlier, we included them in our analysis and named *Ag5r5* and *Ag5r6*, respectively.

Full-length orthologues could be retrieved in most cases from the genomes of anophelines with a few exceptions, some of which representing events of gene gain or gene loss that occurred in single species. This may be the case for *Ag5r4* that was duplicated in *An. funestus* and lost in *An. darlingi*. Moreover, the duplication originating the pair *Ag5r2*/*Ag5r5* most likely took place before anopheline radiation, whereas *Ag5r3* underwent a gene duplication giving rise to *Ag5r6* after the separation of the New World *Nyssorhynchus* species from Old World anophelines, around 100 Mya (Fig. 1). Despite the very large divergence of family members (minimal aa identity 32.9%), the 82 full-length anopheline Ag5 proteins share a common structure, with full conservation of their ten cysteine residues and with Ag5r3/Ag5r6 carrying an additional cysteine pair. Multiple alignment also showed that Ag5r3 deduced proteins carry a conserved DPGR tetrapeptide, previously recognized as of crucial importance for thrombin recognition in an in vitro selection study [70] and shown to occupy the active site cleft of the enzyme in the crystal of the *An. albimanus* anophelin interacting with alpha-thrombin [71]. This tetrapeptide is conserved also in some Ag5r6 proteins, whereas in the remaining ones the R was replaced by a K (Additional file 7). The presence and the conservation of the DPGR motif is intriguing and a possible antithrombin function of Ag5r3 proteins cannot be ruled out, although the expression at similar levels in multiple tissues of both sexes (salivary glands, midgut, malpighian tubes) found by Baker et al. [46] in *An. gambiae* seems to make unlikely this hypothesis. Due to the variety of functions accomplished by members of this family it is difficult to predict or assign possible roles to these anopheline Ag5 salivary proteins. Nevertheless, as far as we know, the best candidates for a blood feeding role may be the gVAG proteins, due to the higher FSG/MSG ratio [20], followed by Ag5r2 and Ag5r4 that still reach relatively high expression in the FSG of *An. gambiae* [46]. Phylogenetic analysis including the 82 Ag5 anopheline proteins identified here yielded four very well supported clades including gVAG, Ag5r4 and the two pairs of duplicated genes Ag5r2/Ag5r5 and Ag5r3/Ag5r6 (Additional file 8).

#### D7 family

The *D7* it is certainly one of the best-known salivary multigene families from blood sucking insects. The first D7 family member was identified 25 years ago in the mosquito *Ae. aegypti* as one of the most abundant proteins found in the saliva of adult females [72]. Following this initial observation the *D7* was shown to be a multigene family in *An. gambiae* [18, 34, 36, 73, 74], to be part of the Odorant Binding Protein (OBP) superfamily [75] and to be widely spread among blood feeding nematocera, with representatives not only in anopheline and culicine mosquitoes [19, 21, 23, 29, 76, 77] but also in sand flies, black flies, frog biting flies and culicoides [22, 78–80]. We will mainly focus here on the *D7* family in anophelines; a comprehensive discussion on the evolution of the D7 protein family in blood feeding insects has been provided elsewhere [6, 22].

**Fig. 2:**
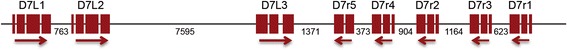
Genomic organization of the *An. gambiae D7* cluster. Schematic representation of the ~20 kb genomic locus on 3R-30B carrying the three long *D7* genes followed by the five short *D7* in the reverse orientation. The *red* boxes represent exons. Introns and intergenic regions are shown as a *black line*. The *red arrows* point to the direction of transcription and numbers indicate the length in bp of the intergenic regions

Mosquito protein family members can be distinguished in short and long D7, possessing one and two OBP domains, respectively. These are atypical because they have seven alpha helices, two additional in comparison to canonical OBPs [81, 82]. The *An. gambiae* genome carries eight members of the *D7* gene family clustered in a region of ~20 kb on 3R-30B. Three genes encode long D7 proteins of ~31–35 kDa (*D7L1*, *D7L2* and *D7L3*) and five code for short D7 proteins of ~17 kDa (*D7r1*, *D7r2*, *D7r3*, *D7r4* and *D7r5*) [18]. The eight genes are organized in two cassettes spaced by a region of ~1.4 kb. The long *D7* cassette includes the three genes placed one after the other in the forward orientation, with *D7L1* and *D7L2* having four exons and *D7L3* with only three exons. The short *D7* cassette carries the five short-form genes in the reverse orientation, with the first four having three exons and the fifth, *D7r5*, having only two exons (Fig. 2). In *An. gambiae* the D7 proteins are among the most abundant salivary components and are specifically expressed in adult female salivary glands [18, 46, 73]. According to transcript representation in sialotranscriptome studies the first four short *D7* (*D7r1*-*D7r4*) appear the most abundantly expressed, whereas *D7L1* and *D7L2* are expressed at lower levels. *D7L3* and *D7r5*, which are the last gene in each cassette, are only poorly transcribed and it was proposed they may be turning into pseudogenes [18, 81, 83].

The functional role of insect OBPs is to bind and carry small hydrophobic compounds such as odorants and pheromones. For this reason it was proposed that the mosquito D7 proteins may help blood feeding by capturing agonists of the hemostatic or inflammatory response of the host [73]. This prediction was confirmed by studies on the *An. stephensi* D7r1, which was named hamadarin [84], and on the *An. gambiae* D7r proteins [83]. Hamadarin was shown to inhibit activation of the plasma contact system and bradykinin release by binding factorXII and high molecular weight kininogen; it may facilitate blood feeding in virtue of its anticlotting and antiinflammatory action [84]. Afterwards, the five *An. gambiae* short D7 were expressed in recombinant form and, with the exception of D7r5, were all shown to bind serotonin (5-HT) and other biogenic amines as histamine (H), epinephrine (E) and norepinephrine (NE), although with some difference in preference and affinity. Biogenic amines, released by platelets and mast cells, elicit pain responses and trigger platelet aggregation, vasoconstriction and inflammation and their sequestration by salivary proteins of blood feeding arthropods seems to be a conserved mechanism of highly adaptive value in the evolution of hematophagy in insects [6]. The *An. gambiae* D7r1, as its orthologue in *An. stephensi* hamadarin, also showed anticlotting activity in the Activated Partial Thromboplastin Test, whereas no function could be assigned so far to the D7r5 protein [83]. Structural and functional analyses of mosquito long D7 proteins, which carry two OBP domains, indicated that they are bifunctional, being able to bind and neutralize two different classes of inflammatory mediators. In fact a long D7 protein from *Ae. aegypti* was shown to bind bioactive lipids, namely cysteinyl leukotrienes (cysLTs), with its N-terminal OBP domain and biogenic amines, as is the case for the *An. gambiae* D7r proteins, with the C-terminal domain [81–83]. CysLTs released by mast cells are potent vasodilators and additionally activate the endothelium inducing swelling, erythema, pain and itching, which may trigger defensive behaviors by the host; therefore, antagonizing their effects may be crucial to guarantee an efficient and uninterrupted blood feeding [6, 81]. Interestingly, the N-terminal OBP domain of the *An. stephensi* D7L1 retained the ability to bind cysLTs but also acquired the capacity to bind thromboxane A2 (TXA2), which stimulates platelet aggregation and it is a powerful vasoconstrictor. On the contrary the C-terminal domain is rearranged in comparison to the *Ae. aegypti* long D7 and to the *An. gambiae* D7r and lost the ability to bind biogenic amines, although it is not yet known if it acquired novel binding capacities [85]. Overall the mosquito D7 family is a very nice paradigm of how gene duplication and divergence, including domain duplications, played a pivotal role in evolution of novel functions and adaptation to hematophagy. This family also illustrates very well a recurrent strategy used by blood feeding arthropods, which is producing large amounts of salivary proteins with high affinity binding activity toward agonist of the host hemostatic and inflammatory responses. The name kratagonist has been proposed for this kind of inhibitors that include, besides the mosquito D7, also lipocalin family members from *Rhodnius prolixus* (binding H, nitric oxide and ADP) and from ticks (binding H, 5-HT, NE, TXA2, cysLTs) as well as sand fly salivary members of the Yellow family, that were also shown to bind 5-HT, E and NE [6, 86].

One hundred twenty seven full-length *D7* family members were retrieved from the genomes of the 19 anophelines analyzed here, 83 being short *D7* and 44 long *D7* (Additional file 2). Identification of orthologues was guided mainly by the gene order in the cluster and partly by sequence similarity to the *An. gambiae* prototypes. However, in a few cases proper assignment was complicated: for this reason in Additional file 2 three short D7 from *An. atroparvus*, *An. albimanus* and *An. darlingi* were indicated as *D7r2*/*D7r3* and one short D7 from *An. farauti* was named as *D7r4*/*D7r2-like.* Overall, with a few exceptions most likely due to incomplete genome assemblies, three short (*D7r1*, *D7r3* and *D7r5*) and two long *D7* genes (*D7L2* and *D7L3*) were found in all anophelines analyzed (Fig. 1) suggesting that the progenitor of anophelines may have carried a cluster of 5 genes. *D7r2* and *D7r4* were absent in representatives of the *Anopheles* and *Nyssorhynchus* subgenera, and *D7r4* was also absent in *An. farauti* and *An. dirus* (*Cellia* subgenus, Neomyzomyia series). It is possible that these two short *D7* may have originated sometime in a progenitor of *Cellia* species by gene duplication of *D7r3* and *D7r1*, respectively. *D7L1* was absent in *An. albimanus* and *An. darlingi* as well as in a few additional anophelines: this distribution would be compatible with its appearance, most likely by duplication of *D7L2*, after the separation of the New World *Nyssorhynchus* species from Old World anophelines, around 100 Mya, followed by sporadic events of gene loss in species belonging to the *Cellia* subgenus (Fig. 1). *An. albimanus* and *An. darlingi* have orthologues of *D7r3* and *D7r5* and three copies each of shorter *D7r* genes typical of *Nyssorhynchus* that appear more closely related to *D7r1* and are most likely the result of multiple gene duplication. We could find only one of these three genes in the genome of *An. darlingi*. However, two additional short *D7* typical of *Nyssorhynchus* were previously found by transcriptome studies: GI: 208657479 (version ACI30036) and GI:208657495 (version ACI30044) [21]. These two additional genes are not included in Additional file 2 but were inserted in Fig. 1 as well as in the following phylogenetic analysis.

**Fig. 3:**
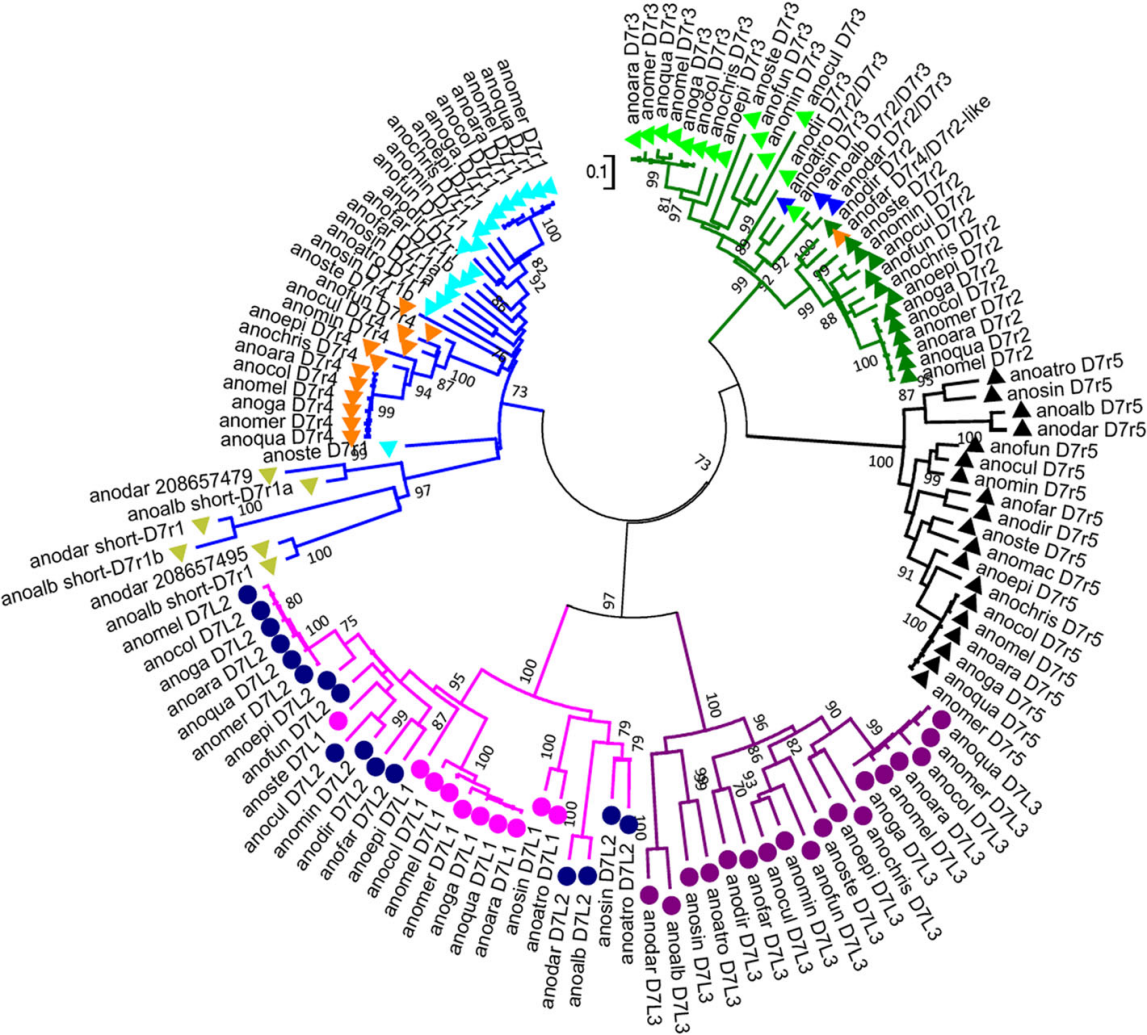
Phylogram of the salivary D7 proteins from anopheline mosquitoes. Numbers in the phylogram nodes show the percent bootstrap support for the phylogeny (≥70%). The bar at the bottom indicates 10% aminoacid divergence in the sequences. Coloured dots and triangles mark D7 long and D7 short proteins, respectively. Also the different clades are in colours: D7r1/D7r4 clade (light blue), D7r2/D7r3 (green), D7r5 (black), D7L1/D7L2 (pink), D7L3 (purple)

Multiple alignment of the 129 D7 family members indicated that D7r proteins align to the C-terminal region of long D7 with good conservation of the Cys framework (not shown). The phylogenetic analysis showed three main well supported clades (Fig. 3): (i) the first includes all D7r1 and D7r4 proteins, with the shorter D7 typical of *Nyssorhynchus* forming a strongly supported subclade; (ii) the second groups all D7 long proteins with two independent subclades, one comprising all D7L1 and D7L2 and the other the D7L3 proteins; (iii) the third clade includes D7r5 proteins, which are part of a separated and well supported subclade, as well as D7r2 and D7r3 proteins. This distribution fully agrees with the interpretation pointing to the pairs *D7L1*/*D7L2*, *D7r1*/*D7r4* and *D7r2*/*D7r3* as the products of gene duplication.

#### 30 kDa family

Members of the 30 kDa family (also sometime indicated as 30 kDa allergens or GE-rich proteins) were first described as salivary allergens of *Aedes* mosquitoes [87]. With the growing of sialotranscriptome studies members of the family were found in culicine and anopheline mosquitoes [18, 19, 23, 29, 55, 74, 76, 77, 88, 89], but also in black flies [90], and more distantly related family members were recognized in sand flies [22]. 30 kDa proteins from *Ae. aegypti* (aegyptin) and *An. stephensi* (anopheline anti-platelet protein, aapp) were shown to have a conserved function and to inhibit collagen-induced platelet aggregation by binding to collagen and preventing its interaction with glycoprotein VI (GPVI), integrin α2β1 and von Willebrand Factor (vWf) [91, 92]. 30 kDa proteins are abundantly and specifically expressed in mosquito female salivary glands [18, 20, 30] and the promoter of the *An. stephensi* gene was shown to drive strong tissue-specific expression of exogenous genes in the female salivary glands of transgenic anophelines [93]. Noteworthy, anopheline mosquitoes, as also confirmed here, carry a single gene belonging to the family while culicine mosquitoes have multiple copies. As previously described, mature 30 kDa family proteins are characterized by two distinct domain: the N-terminal half, highly acidic and of low complexity (being rich in Gly, Glu and Asp residues), and the C-terminal domain, more complex and carrying four conserved cysteines [91, 92]. The C-terminal domain, which consists mainly of two alpha helices spaced by a short loop and connected by the two conserved disulfide bridges [94], has been shown to be the main region of the aegyptin involved in the binding to collagen [95].

In *An. gambiae* the 30 kDa protein is encoded by a four-exon gene (AGAP009974) located on 3R-36B. Full-length orthologues were retrieved in all anophelines (Fig. 1) and showed a degree of amino acid identity in the range of 46.7 to 81%. The mature proteins vary from 217 (*An. darlingi*) to 271 (*An. farauti*) amino acids in length, with isoelectric points between 3.9 and 4.3 (Additional file 2). Multiple alignment of the deduced proteins showed that the N-terminal low complexity domain is 96 to 153 amino acids in length and it is the region responsible both for the size heterogeneity and for the acidic nature of the protein, being highly enriched in Asp, Glu and Gly residues (54.6–71.5%) with 31–53 negatively charged amino acids against 1–6 positively charged (Additional file 9). The C-terminal domain carrying the four cysteines is conserved both in size (119–121 aa) and in sequence and it is essentially neutral with a difference in charged residues between −1 and +4. Phylogenetic analysis of anopheline 30 kDa family members showed a clustering fully consistent with known relationships between anopheline mosquitoes (not shown). A more comprehensive bootstrapped phylogram including Anopheline, Culicine, Simulium and Phlebotomus sequences has been reported previously [22].

### Conserved mosquito families

Within this group are included protein families that are found in the saliva of both anopheline and culicine mosquitoes but were not detected so far in the saliva of other blood feeding arthropods. It consist mainly of single copy genes (SG5, SG8, SG9/41kDda, 55.3 kDa) but includes also the highly divergent multicopy 37.7 kDa family as well as the large SG1 family.

#### Hypothetical 37.7 kDa family

**Fig. 4:**
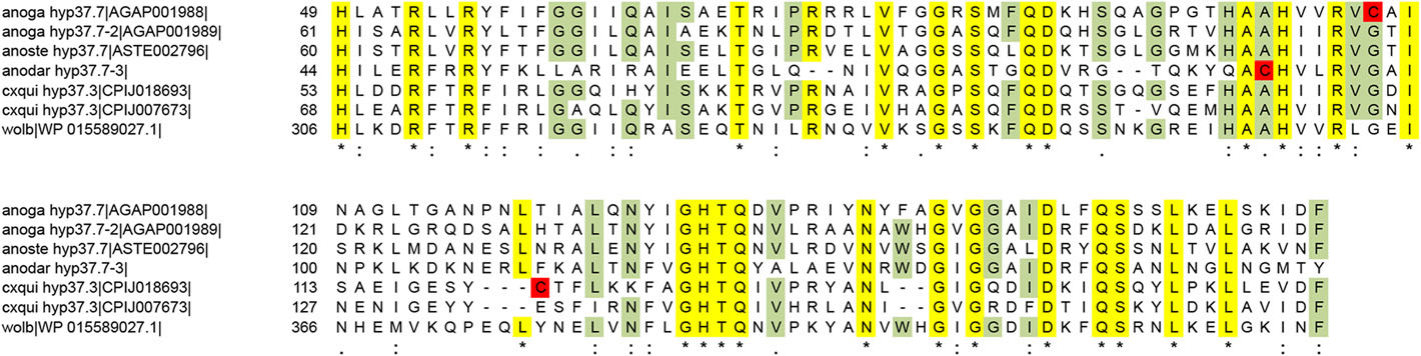
Alignment of mosquito hyp37.7 kDa family members to an hypothetical protein from *Wolbachia*. Multiple alignment of hyp37.7 family members from *An. gambiae*, *An. stephensi*, *An. darlingi* and *Cx. quinquefasciatus* to an hypothetical protein from a *Wolbachia* endosimbiont of *Drosophila simulans*. Only the conserved 115 amino acid region is shown. Fully conserved residues (*yellow*), cysteins (*red*) and residues conserved in at least 2/3 of the aligned sequences (*green*) are highlighted. Numbers indicate aminoacid positions. Mosquito species names are abbreviated with the first letters of the generic name and the first three letters of the specific name; wolb indicates the *Wolbachia* sequence. Accession numbers follow (when available)

The first member of this family, encoding a protein of 37.3 kDa, was identified in *An. stephensi* [23], and a putative orthologue was found later in *An. gambiae* [18]. In the African malaria mosquito there are actually two family members: *hyp37.7* (AGAP001988) and *hyp37.7-2* (AGAP001989) located on 2R-10A and separated by a short intergenic region of approximately 0.5 kb. They are intronless and encode putative secreted proteins as indicated by signal peptide prediction analysis (Additional file 2). Considering their high divergence (the encoded proteins show 39% identity and 53% similarity) they are most likely the result of an ancient gene duplication and are highly or specifically expressed in female salivary glands [18, 46]. Blastp searches using the *An. gambiae* hyp37.7 or hyp37.7-2 retrieve proteins from other anophelines, two hyp37.3 salivary proteins from *Culex quinquefasciatus* (CPIJ018693, CPIJ018673), two hypothetical proteins from *Aedes albopictus* (AALF003062, AALF000758) but also an hypothetical protein from a *Wolbachia* endosimbiont of *Drosophila simulans* (WP 015589027.1). This *Wolbachia* protein shows 49% identity and 61% similarity to hyp37.7 (43% query cover, *E*-value 5e-24) and 36% identity and 56% similarity to hyp37.7-2 (88% query cover, *E*-value 1e-42). This similarity to a *Wolbachia* protein, along with the single exon structure of *hyp37.7* family members, suggest that this gene family may have originated by horizontal transfer from the genome of a mosquito endosymbiont, as previously proposed for other *An. gambiae* salivary transcribed genes [18]. Multiple alignment of the mosquito and *Wolbachia* proteins (excluding the *Ae. albopictus* AALF003062 and AALF000758 for which there is no evidence of salivary glands expression) shows a well conserved region of 115 amino acids in length (Fig. 4).

A total of 40 complete *hyp37.7* family members were found in the nineteen anopheline species considered here, with at least one representative per species and with most of the family members (33/40) predicted to encode a secretory signal peptide (Fig. 1, Additional file 2). The *hyp37.7* appears to be both a highly divergent and a highly dynamic gene family. The amino acid identity among different family members is in the range of 16 to 88% (hyp37.7, 38–72%; hyp37.7-2, 16–78%; hyp37.7-3, 23–88%). In addition, there is a large variation in gene copy numbers, indicative of multiple events of gene gain/loss with a few species carrying just one family member (*An. atroparvus* and *An. sinensis*, subgenus *Anopheles*), most species having two (all species of the series Pyrethophorus and Neocellia, subgenus *Cellia*) while the remaining possess three to five copies (Fig. 1). The function of members of this family is presently unknown.

#### SG1 family

In *An. gambiae* 7 members of the SG1 family have been recognized [18, 35, 36, 74]. Five of them, named *SG1* (AGAP000612), *SG1a* (AGAP000611), *Saglin* (AGAP000610), *SG1-like2* (AGAP000609) and *SG1-like3* (AGAP000607), are closely clustered in a ~10 kb region on the X chromosome, division 1D (Additional file 10A). A sixth member, *SG1b* (AGAP000549), is located on the same chromosomal division at a distance of approximately 1.2 Mb, whereas the last gene, named *TRIO* (AGAP001374), is located on 2R-8A. Remarkably, gene family members are all intronless, unusual for eukaryotic genes coding for these relatively large proteins (~45 kDa), suggesting possible acquisition by horizontal transfer. They all share a very similar expression profile with strong upregulation in FSG [18, 20, 35, 36, 46] and a likely physiological role connected to blood feeding. *An. gambiae* SG1 family members are very divergent with a minimum amino acid identity of ~14% and a maximum identity of ~30–31% among SG1, SG1a and Saglin. PSIblast search with the *An. gambiae* SG1 protein against the non redundant database allowed to retrieve all anopheline SG1 family members but in addition, after a few iteration, also the salivary 62 kDa proteins of *Aedes* mosquitoes [22]. The *Anopheles* SG1 and the *Aedes* 62 kDa families are very distantly related (protein identity in the range of 11–17%) but should be considered as part of the same superfamily. According to these considerations the *SG1* family, initially indicated as unique to anophelines [18], was reclassified as part of the *SG1/62 kDa* superfamily of mosquitoes [22] and it is included here among the group of the conserved mosquito family.

**Fig. 5:**
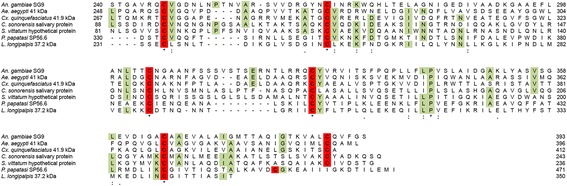
Alignment of the C-terminal region of selected members of the expanded SG9/41 kDa family. Multiple alignment of the C-terminal region encompassing ~200 amino acids of members of the SG9/41 kDa family from a few representative species. Residues conserved in at > 50% of the aligned sequences are highlighted in *green*, cysteines are shown in red. Accession numbers: *Anopheles gambiae* gi:347971052, *Aedes aegypti* gi:94468848, *Culex quinquefasciatus* gi:38350631, *Culicoides sonorensis* gi:51557691, *Simulium vittatum* gi:197260858, *Phlebotomus papatasi* gi:449060677, *Lutzomyia longipalpis* gi:42491533

The *SG1* family appeared well conserved among species of the subgenus *Cellia*, where seven family members, organized similarly to *An. gambiae*, could be easily recognized*.* The *SG1a* gene was missing in *An. atroparvus* and *An. sinensis* (subgenus *Anopheles*) as well as in *An. albimanus* (subgenus *Nyssorhynchous*), who also lacked *SG1*. Therefore, these species carry a cluster of four and three genes, respectively, rather than five genes as in the *Cellia* species (Figs. 1 and 5). *An. darlingi* was not readily assessable since the *SG1* cluster, consisting of three to five genes in the other anophelines, could not be retrieved from the genome of this species and only *SG1b* and *TRIO* were identified. However, *An. darlingi* possesses at least two additional family members, i.e. *Saglin* (ACI30180) and *SG1-like3* (ACI30121, ACI30123) as indicated by a previous transcriptome analysis [21]. Overall 119 full-length *SG1* family members were identified in the genomes of the 19 anophelines studied here, and most (109/119) are predicted to encode proteins carrying a signal peptide at their N-terminus (Additional file 2), which is in agreement with the evidence for secretion found for the *An. gambiae* SG1b, SG1 and SG1-like3 by Edman degradation of SDS-PAGE protein bands [74]. According to the distribution of family members among anophelines, to sequence comparison and phylogenetic analysis a possible scenario is that *Saglin*, *SG1-like2*, *SG1-like3*, *SG1b* and *TRIO* were already present more than 100 Mya when anopheline radiation is supposed to have started [31], as indicated by their presence in the genome of all species of the three subgenera considered here (Fig. 1). *SG1* may have evolved from *Saglin* by gene duplication after separation of Old World anophelines from New World species. A second gene duplication, which may have taken place in the progenitor of *Cellia*, gave rise to *SG1a* (either from *Saglin* or from *SG1*). An alternative, less conservative explanation of the situation observed today would be independent gene loss events in *An. albimanus*, *An. atroparvus* and *An. sinensis*.

Due to the absence of significant similarity to known proteins the function of members of the *SG1* family is still unknown. However, Saglin was suggested to be involved in *Anopheles* salivary gland invasion by *Plasmodium* sporozoites [96] and its downregulation by RNAi drastically reduced the number of *P. falciparum* sporozoites in the salivary glands of infected *An. gambiae* [97]. Notably, *Saglin* was found in the genome of all nineteen anopheline species analyzed here, many of which are good malaria vectors. The functional significance of the expansion of the *SG1* family and the possible involvement of other family members in pathogen-vector interaction stays as an open question. However, the ability of the SM1 peptide to interact not only to Saglin but also to SG1, as shown by cross-linking experiments [97], raises the possibility that other family members may play some role in *Plasmodium* salivary gland invasion. A phylogram of the anopheline SG1 protein family is included as supplemental material (Additional file 10B).

#### SG5, SG8 and SG9/41 kDa families

Founders of the *SG5*, *SG8* and *SG9* families of anophelines were identified during a signal sequence trap screening in *An. gambiae* [36]. Full-length orthologues of *gSG5*, *gSG8* and *gSG9* were retrieved in all anophelines (Fig. 1, Additional file 2) and members of the same families were also identified in sialotranscriptomes of culicine mosquitoes, where the *SG9* was named as *41 kDa* family [29, 76]. In *An. gambiae* and *Ae. aegypti SG5* and *SG8* transcripts were found specifically expressed in FSG whereas *SG9*/*41 kDa* family members were expressed in both FSG and MSG [18, 20, 30, 46, 76]. Conservation of the SG5 and SG8 protein families among anophelines is in the range of 48–87% and 50–78%, respectively. When the *An. gambiae* and *Ae. aegypti* proteins are compared conservation drops (SG5: 27% id., 54% sim.; SG8: 34% id., 66% sim.), nevertheless, multiple alignments show preservation of the overall structure with full conservation of the 8 (SG5) and 7 cysteine residues (SG8) (not shown). The function of SG5 and SG8 family members is presently unknown, but considering their overexpression in FSG they are expected to affect blood feeding/host physiology.

Also the SG9/41 kDa protein family appears well conserved among anophelines (50–78% identity). Comparison with the NR protein database only retrieves mosquito family members from *Ae. aegypti*, *Cx. quinquefasciatus* and *Ae. albopictus*; moreover a member of the family was also reported in the non-blood feeding mosquito *Toxorhinchites amboinensis* [98]. However, use of PSI-BLAST allowed to retrieve after a few iterations, first an unknown salivary protein from *Culicoides sonorensis*, then an hypothetical protein from *Simulium vittatum* and finally a few salivary proteins from sand flies (39 kDa protein from *Phlebotomus ariasi*, SP56.6 from *P. papatasi*, 37.2 kDa from *Lutzomyia longipalpis* and SP19 from *P. perniciosus*). Despite the different sizes (from the 236 aa of the *S. vittatum* protein to the 471 aa of the *P. papatasi* SP56.6) and the divergence in the N-terminal region, multiple alignment of SG9/41 kDa family members from a few representative species shows a good conservation of the C-terminal region encompassing ~200 amino acids and carrying six fully conserved cysteine residues (Fig. 5). These observations suggest that this protein family, still classified here as conserved mosquito family, may be more widely spread among blood feeding Nematocera than previously thought. The function of members of the SG9/41 kDa family is presently unknown. A phylogram including this expanded SG9/41 kDa protein family has been previously reported [21].

#### 55.3 kDa family

The *An. gambiae* 55.3 kDa salivary protein is encoded by AGAP005822, an intronless gene located on 2 L-23A. Both according to RT-PCR and microarray data it is specifically expressed in adult salivary glands of both sexes [18, 20, 46]. Orthologues are also present in culicine mosquitoes, where they are slightly larger in size and, therefore, were classified as 56.5 kDa proteins. They have an expression pattern similar to the anopheline 55.3 kDa proteins [29, 76, 77], although a recent RNA-Seq analysis in *Ae. aegypti* showed significantly higher expression in male salivary glands as compared to female glands [30]. Database searches using the *An. gambiae* protein only retrieve mosquito proteins; however, after a few iterations of PSIblast also bacterial proteins start to appear. This observation, joined to the uniexonic structure, led to the suggestion that this gene family may have been acquired by mosquitoes through horizontal transfer from some bacterial genome [22]. Orthologues, mostly full-length, were retrieved from all anopheline genomes (Fig. 1, Additional file 2). Multiple alignment of anopheline and culicine members of the 55.3 kDa/56.5 kDa family shows a highly conserved block of 27 aminoacids at the N-terminus (pattern: RxV[LM]DSLVE[STVA]GSPIFQ[GAS]L[SA]N[AV]A[RK][LI]S[ST]G) and six fully conserved Cys residues at the C-terminus (Additional file 11), with amino acid identities in the range of 55–86% between anopheline family members and 31–37% between anophelines and culicines. Secondary structure prediction suggests that proteins of this family, whose function is presently unknown, may have a high alpha helical content.

### Anopheline-specific families

We describe here a few genes that have been found so far only in the saliva of anopheline mosquitoes and, therefore, should have evolved in the Anopheles genus not earlier than ~145 Mya, after that ancestral anophelines diverged from ancestral culicines [57]. Some of them, as the *SG2* and *SG6* families, are absent in the Neotropical species *An. albimanus* and *An. darlingi* (Fig. 1) and therefore should have appeared not earlier than ~100 Mya, when South America started to separate from Africa. Other evolved even later during anopheline radiation as is the case of the *hyp10*/*hyp12* family, that is absent in species of the genera *Anopheles* and *Nyssorhynchus* and most likely originated sometime in a progenitor of the *Cellia* subgenus.

#### cE5/anophelin family

The first member of the cE5/anophelin family was identified in *An. gambiae* as a secreted salivary component with no similarity to other known polypeptides and named cE5 [35]. Shortly later, a salivary inhibitor of thrombin from the South American malaria vector *An. albimanus* was biochemically identified and named anophelin. cDNA cloning and sequencing indicated the orthology relationships between these two salivary proteins [99]. Kinetic and structural studies showed that cE5/anophelin family members are intrinsically disordered, tight-binding reversible inhibitors with a unique mechanism of thrombin binding [71, 100, 101]. In *An. gambiae* the *cE5* transcript appeared expressed at high levels in female salivary glands but also, and surprisingly for a thrombin inhibitor, in several additional tissues [35, 46]. However, the corresponding protein product was only detected in adult female salivary glands suggesting that some post-transcriptional mechanism of gene regulation is involved in the sex- and tissue-specific protein translation [101]. Members of the family were present in all species of the three subgenera *Cellia*, *Anopheles* and *Nyssorhynchus* included in the 16 anopheline genome project (Fig. 1, Additional file 2). Alignment of the different family members shows a largely conserved acidic N-terminal block of sixteen amino acids, with a consensus APQY[AST]xG[DE]xP[ST]YD[DE][DE][DET], and a highly conserved DPGR tetrapeptide toward the C-terminus (only exceptions the *An. epiroticus* and *An. atroparvus* proteins where Pro is replaced by Ala). Intriguingly, this tetrapeptide had been previously recognized in an in vitro selection study as crucial for αlpha-thrombin recognition [70]. The two conserved blocks are spaced by a more divergent central region of 31–40 aminoacids made up for approximately one third of its length of acidic residues (D or E). The alignment also shows that family members from the New World *Nyssorhynchus* species *An. albimanus* and *An. darlingi* are slightly shorter than in other anopheline species, which carry an additional stretch of 7 to 21 amino acids enriched in serine (Additional file 12). Moreover, most members of the *An. gambiae* complex carry at the N-terminus the RGD tripeptide known for the ability to bind integrins, although it is not flanked by the typical pair of cysteines involved in a disulphide bond [59]. The cE5/anophelin family looks to be quite variable with an identity range of 31.5 to 65.7% among all anophelines (44.9 to 65.7% within the subgenus *Cellia*). The *An. gambiae* cE5 protein was shown to be antigenic to humans and there is evidence it may be useful as a tool to evaluate efficacy of insecticide-treated bednets in reducing human-vector contact [102].

#### Hypothetical 4.2 and hypothetical 13

Transcripts encoding hyp4.2 (AGAP003473) and hyp13 (AGAP003474) were identified in *An. gambiae* where the corresponding genes are located close to each other on 2R:15C at a distance of ~1.6 kb. They have a very similar, almost ubiquitous, expression pattern with higher levels in both male and female adult glands [18, 46]. *Hyp4.2* and *hyp13* code for mature peptides of ~4.3 kDa (40 aa) and ~3.8 kDa (34 aa), with 35% identity and 45% similarity. The similar structure, expression and close proximity suggest they may be the result of an old gene duplication. Clear orthologues of *hyp4.2* and *hyp13* could be identified only in the Pyretophora series (i.e. the *An. gambiae* species complex, *An. christyi* and *An. epiroticus*) where they share 39–62% (hyp4.2) and 41–59% (hyp13) amino acid identity. It is possible that orthologues are present in the other anophelines but difficult to reliably identify because of the combination of short size and wide divergence. Their function is presently unknown.

#### Hypothetical 6.2 and hypothetical 8.2

**Fig. 6:**
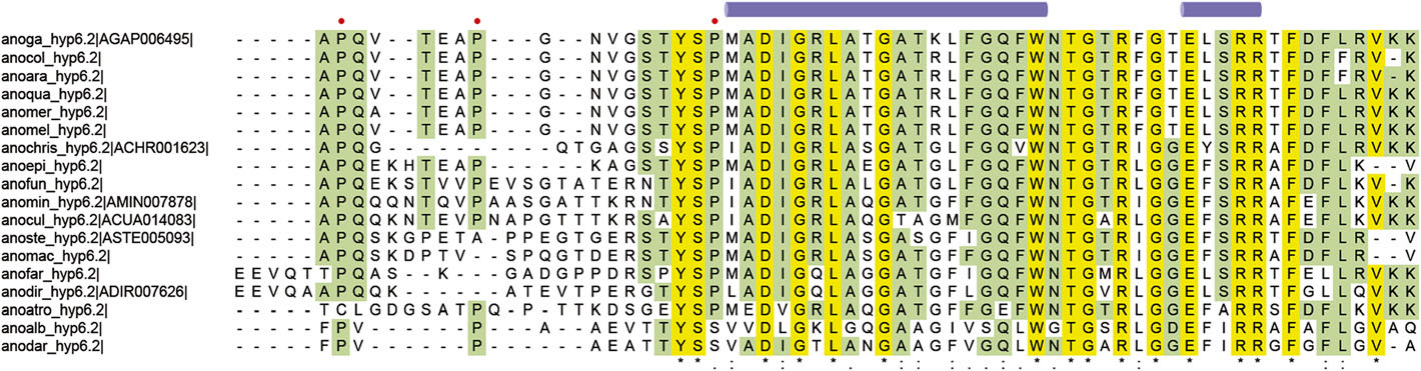
Alignment of anopheline hyp6.2 proteins. Multiple alignment of hyp6.2 family members from 18 anopheline species. Invariant positions are highlighted in *yellow* and residues conserved in at least two thirds of the aligned sequences in green. Predicted alpha helices in the middle and C-terminal regions (*blue cylinders*) and conserved prolines in the amino-terminal region (*red dots*) are shown above the alignment. Species names are abbreviated with the first three letters of the generic name and the first three-four letters of the specific name. VectorBase accession numbers follow (when available)

*Hyp6.2* and *hyp8.2* were found highly enriched in *An. gambiae* female salivary glands [18, 20, 46] and their salivary expression was confirmed by sialotranscriptome studies in a few additional anopheline species [19, 21]. In *An. gambiae* hyp6.2 and hyp8.2 are encoded by two intronless genes located at a distance of ~1.5 kb on 2 L-25A, a division where also other salivary genes are located (SG2, SG2b, SG3). Apparently they seem the result of a gene duplication, although these two small proteins do not share significant sequence similarity. Mature hyp6.2 and hyp8.2 have molecular weights of ~6.2 kDa (58 aa) and ~7.9 kDa (73 aa), do not carry any cysteine and are differently charged, with hyp6.2 being basic (pI 10.4) and hyp8.2 acidic (pI 4.2). Database searches do not show similarity to any known protein and, although their pattern of expression suggest a possible role in blood feeding, their function is presently unknown. Full-length orthologues of *hyp6.2* and *hyp8.2* were found in all anopheline species but *An. sinensis* where only a partial *hyp6.2* could be retrieved (Fig. 1, Additional file 2). Multiple alignment of anopheline hyp6.2 proteins shows remarkable conservation of middle and C-terminal regions, which include 44 aminoacids with 16 invariant positions and it is predicted to structure forming two alpha helices. The aminoterminal region appears less preserved and, as previously noted [19], has three conserved prolines probably making two loops with variable lengths (Fig. 6). On the contrary alignment of hyp8.2 proteins showed an unusually large divergence between species with no invariant positions and amino acid identity varying in a wide range (12.2 to 68.1%) in pairwise comparisons.

#### Hypothetical 10 and hypothetical 12

In *An. gambiae hyp10* and *hyp12* are expressed in the salivary glands of both adult males and females [18, 20] and encode putative mature polypeptides of 67 (7.5 kDa) and 71 (7.9 kDa) amino acids in length, respectively. They are located on 3R-30B, the same chromosomal division where the D7 cluster maps, and are arranged in tandem and separated by a ~1.2 kb intergenic region. The two predicted polypeptides are 43% identical (60% similar) and are clearly the products of a gene duplication. They are absent in species of the subgenera *Anopheles* and *Nyssorhynchus*, but were found in all species of the subgenus *Cellia* analyzed in this study (only exception *An. maculatus* where, because of the short genomic contig, only a truncated hyp10 was found). The tandem arrangement observed in *An. gambiae* is conserved in the other *Cellia* where these two small genes are spaced by 0.5–1.8 kb intergenic regions. Alignment of putative mature polypeptides highlights the four highly conserved cysteines, which indicates they must share similar folding. Hyp10 shows 44–76% amino acid identity in the different species and similar values were found for hyp12 (41–77%); the two paralogues, as expected, are more distantly related with identities ranging within *Cellia* from 34 to 53%. Secondary structure prediction analysis indicated that hyp10/hyp12 family members have a secondary structure characterized by two alpha-helices (N-terminal and C-terminal) separated by a central loop 5–11 aa in length, suggesting a simple structure where the two helices may be hold together by disulfide bridges (Additional file 13A). These two proteins, as mentioned above, are restricted to anophelines of the subgenus *Cellia*, do not show similarity to any known protein and their function is presently unknown, although their presence in the saliva of both adult male and females may suggest a potential antimicrobial role. Phylogenetic analysis yielded two well-defined and supported clades for hyp10 and hyp12 (Additional file 13B).

#### Hypothetical 15 and hypothetical 17

**Fig. 7:**
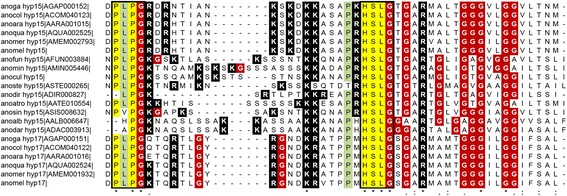
Alignment of the anopheline hyp15/hyp17 family members. Multiple alignment of the anopheline mature hyp15 and hyp17 proteins. Fully conserved residues are indicated by *asterisks*. Positively charged amino acids (K, R) and glycines (G) are shown in *black* and *red* background, respectively. Other residues are highlighted in *yellow* (fully conserved) or *green* (conserved in at least 2/3 of the aligned sequences). The consensus tetrapeptide PLPG at the N-terminus and the conserved tetrapeptide HSLG in the central region are boxed. Species names are abbreviated as in Fig. 6

The hyp15/17 protein family includes a group of small proteins specifically found in anopheline mosquitoes and whose expression in *An. gambiae* is highly enriched in adult female salivary glands [18, 46]. Gene family members encode putative mature polypeptides of 4.1–5.4 kDa (41–56 aa, pI 10–12) characterized by the highly conserved tetrapeptide PLPG at the N-terminus and by the fully conserved tetrapeptide HSLG spaced by a region (14–26 aa) enriched in positively charged residues (K, R). A glycin-rich carboxy terminus (27–36% in the last 21–22 residues) follows the HSLG motif (Fig. 7). Secondary structure prediction analysis suggests that these Cys-free proteins are largely disordered. The function of hyp15/hyp17 salivary proteins is unknown but the expression profile indicates they may be involved in blood feeding, perhaps binding some receptor or hemostasis mediator or containing microbial growth. Database searches only retrieve anopheline family members and do not show significant similarity to other known proteins. At least one member of the family was found in the different subgenera/series represented by the eighteen species studied here. Considering the small length of the gene(s), the inability to retrieve orthologs from the genomes of *An. christyi*, *An. maculatus* and *An. farauti* may be more likely due to incomplete genome assembly in these species rather than to events of gene loss. Noteworthy, full-length orthologues of both family members were found in species of the *An. gambiae* complex, with *An. epiroticus* carrying degenerated copies containing frameshifts, whereas only one member was found in the other anophelines. According to sequence similarity, the most likely scenario is that hyp15 was the ancestor gene and that a duplication took place in the lineage leading to *Pyrethophorus* originating the hyp17. Indeed in *An. gambiae* the two genes are located on chromosome X where they show a tandem arrangement and are separated by a very short intergenic region (~300 bp). Overall, outside the *An. gambiae* complex, hyp15 proteins share 34 to 86% identical amino acid residues among the different species.

#### SG2 family

The founder of this family was initially identified during a signal sequence trap screening in *An. gambiae* and named *gSG2* [35], and a second family member was found during a second round of screening and named *gSG2-like* [36]. *gSG2* (AGAP006506) and *gSG2-like* (AGAP006504) are located on 2 L-25A at a distance of ~5.1 kb; careful examination of the surrounding regions revealed the presence of a third member of the family (AGAP006505) located in between. *SG2-like* has been sometime also indicated as *SG2a* or *SG2A*, which created some confusion; however, following the physical order in the cluster we propose here to name AGAP006505 as *gSG2a* and indicate AGAP006504 as *gSG2b* or *gSG2-like. gSG2* and *gSG2b* were found expressed in both male and female salivary glands [20, 35, 36, 46] and orthologues were identified in sialotranscriptomes of *An. stephensi* [23], *An. funestus* [19] and *An. darlingi* [21]. *gSG2a* most likely originated by gene duplication from *gSG2b* but was neither found during salivary transcriptome analyses nor differentially expressed in salivary glands [46] and for this reason will not be considered here.

The *An. gambiae SG2* and *SG2b* encode putative secreted proteins of 9.7 kDa (94 aa, pI 3.5) and 15.6 kDa (155 aa, pI 7.02) with a limited similarity to each other. They are both low-complexity proteins enriched in Gly (SG2 21.3%, SG2b 27.1%), Phe (18.1%, 11.6%) and Gln (5.3%, 18.1%), and high similarity matches are only produced with other anopheline proteins. Orthologues of both SG2 and SG2b were retrieved from the genomes of most anopheline species analyzed here as reported in Fig. 1. The inability to identify orthologues in *An. darlingi* by genome tblastn searches, despite the fact that at least two *SG2* family members were previously identified during sialotranscriptome analysis [21], suggest that this may be due perhaps to incomplete genome assembly or low-complexity and divergence rather than to gene loss events. The function of members of this family is presently unknown but their Gly-rich composition reminds of antimicrobial peptides isolated from insects [103, 104], suggesting they may be assisting feeding both in males and females acting as antimicrobials.

#### SG6 family

gSG6 is a small protein (mature polypeptide 87 aa) first identified in *An. gambiae* where it is specifically expressed in adult female salivary glands [36]. gSG6 must plays some relevant role in hematophagy since its depletion by RNAi affects mosquito blood feeding ability [105]; nevertheless, its specific function remained elusive so far. The search for orthologues among the anopheline species analyzed here allowed to retrieve SG6 family members in all species of the subgenera *Cellia* and *Anopheles*. However, notably, it was absent both in *An. albimanus* and *An. darlingi* suggesting that, as also previously suggested [105], SG6 was either lost in the progenitor of the New World *Nyssorhynchus* species or appeared later in Old World anophelines. Alignment of the seventeen family members available so far shows a full conservation of the ten Cys residues and an overall good degree of similarity among anophelines (Additional file 14), with a minimum of 52.5% identity and 72% similarity between *An. gambiae* and *An. farauti*. The restriction to anopheline mosquitoes, the absence of significant similarity to any known protein in databases and the antigenic properties of gSG6 allowed to exploit the IgG response to this *An. gambiae* protein as marker of human exposure to bites of Afrotropical malaria vectors (see below).

#### SG7 family

The founder of this family was first identified in *An. gambiae* and named *gSG7* [36] and a second family member, named *gSG7-2* was identified shortly later [18]. These two genes show a tandem arrangement on 3R-30A and are spaced by a short intergenic region (~0.8 kb); they encode mature proteins of approx. 13.5 kDa (118 and 116 amino acids, respectively) that are highly enriched or specifically expressed in the female salivary glands [18, 36, 46]. As summarized in Fig. 1 full-length orthologues of *gSG7* and *gSG7-2* were found in the genomes of most anophelines analyzed here. A third member of the family, which was named *SG7-3*, was found close by in the genomes of *An. gambiae* and *An. coluzzii*; degenerated copies containing frameshifts were also present in *An. quadriannulatus* and *An. merus*, whereas it could not be traced in other anophelines. Sequence comparison and phylogenetic analysis indicates that *SG7-3* most likely originated from *SG7-2* by gene duplication; in *An. gambiae* there is no evidence of *SG7-3* salivary expression, both according to sialotranscriptomes and to microarray analyses, suggesting that either this gene is not expressed at all or may have acquired different tissue-specificity. Alignment of the 37 SG7 family proteins shows a common framework of four highly conserved Cys residues; most SG7-2 proteins and the SG7 of *An. minimus* and *An. culicifacies* carry an additional Cys (Additional file 15A). Sequence comparison and secondary structure prediction analysis suggests that SG7 and SG7-2 proteins have a high alpha helical content with four to five conserved helices. Phylogenetic analysis yielded two well distinct clades, suggesting that *SG7* and *SG7-2* are the products of a gene duplication that predated anopheline radiation (Additional file 15B). A more recent gene duplication may have originated *SG7-3* in members of the *An. gambiae* complex. SG7 proteins share 42–85% identity and SG7-2 family members share 46 to 89% identical amino acid residues, with a minimum overall identity between family members of 29%.

The first member of the family whose function was clarified is the *An. stephensi* SG7, which was named anophensin and shown to inhibit the kallikrein-kinin system and bradykinin release [106]; it is supposed to help mosquito blood feeding in virtue of this antiinflammatory and anticoagulant action. Surprisingly, the SG7 proteins of *An. albimanus* (named albicin) and *An. darlingi* were recently shown to play a different role, being able to inhibit the alternative pathway of complement. On the contrary the *An. albimanus* SG7-2 and the saliva of a few representative anopheline species from the Old World did not, suggesting this is a specific function evolved in the saliva of New World species [107]. Anophensin binds factor XII (FXII) and high molecular weigth kininogen (HK) while albicin binds the C3 convertase enzymatic complex; it is likely that also other members of the *SG7*/*SG7-2* gene family act by binding and inhibiting players of the hemostatic and/or inflammatory response.

**Fig. 8:**
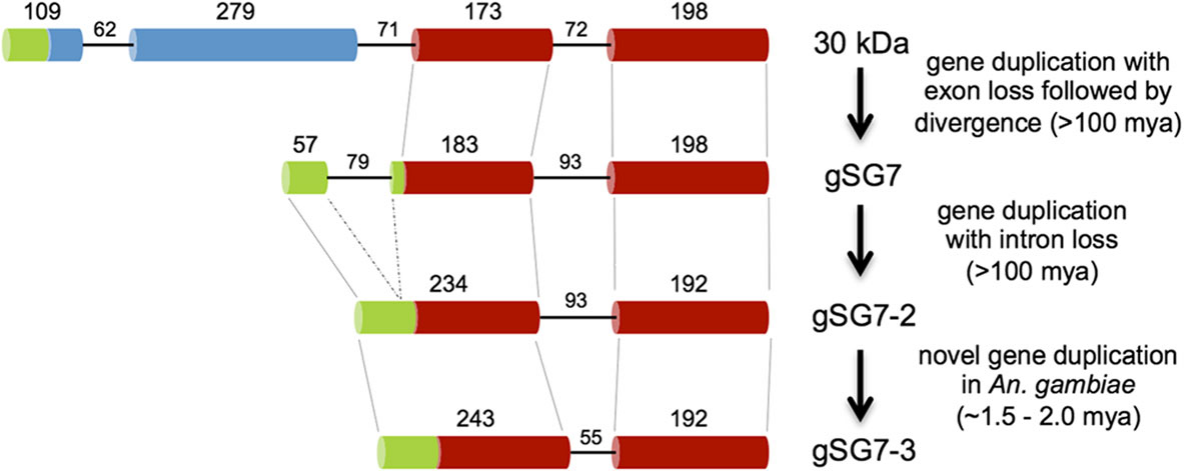
Origin of the *SG7* family from the gene encoding the *An. gambiae* 30 kDa protein. The structure of the *30 kDa*, *gSG7*, *gSG7-2* and *gSG7-3* genes of *An. gambiae* is shown. Exon and introns are represented by boxes and lines, respectively. Numbers indicates the length in base pairs. Conserved exon regions are marked in red and the portion encoding signal peptides in green. The likely scenario leading to the origin of the *SG7* family as we can observe today in *An. gambiae* is shortly described on the right (see main text for additional details)

Interestingly, the *SG7* family offers a nice paradigm of how novel salivary gene functions may evolve. Indeed, blast searches using the *An. gambiae* SG7 protein retrieve with high significance (*E*-values < 6e-12) several anopheline family members but also, with a low significance (*E*-value 0.1, coverage 56%, identity 31%) the 30 kDa salivary protein from *Cx. quinquefasciatus*. Inclusion of this entry in a PSI-blast iteration allowed to retrieve 30 kDa family members from *Ae. aegypti* and *Ae. albopictus* and, after an additional iteration, also several anopheline 30 kDa proteins. As previously reported [31] a careful examination of the genomic loci encoding the *An. gambiae* 30 kDa and SG7 proteins suggested that the anopheline SG7 family of proteins most likely originated from the more ancient *30 kDa*, an “older” gene already present in the blood feeding ancestor of mosquitoes and black flies. The degree of similarity between SG7/SG7-2/SG7-3 and the 30 kDa protein (exons 3–4) is rather low (in *An. gambiae* 23–28% identity, 44–47% similarity). However, the conserved Cys residues, predicted secondary structure and intron/exon boundaries support this scenario. The first member of the family was most likely *SG7* that has a three-exon structure and may have arisen from the the 30 kDa protein by loss of the exon 2, which encodes the highly acidic and low complexity region enriched in Gly, Glu and Asp residues (Fig. 8, Additional file 15C). Therefore, the “novel” SG7 protein retained the region corresponding to the C-terminal half of the 30 kDa protein, that is encoded by exons three and four and it is the portion responsible for the binding to collagen [95]. The SG7 protein appeared sometime in the progenitor of anopheline mosquitoes after the separation from culicines and then could start to diverge acquiring new binding properties and novel functions, as shown by anophensin and albicin today (see above). Afterwards, duplication of *SG7* with loss of the first intron generated *SG7-2*, an event that certainly took place around 100 mya or more, i.e. before separation of Old World mosquitoes from New World ones. This determined the tandem arrangement of *SG7* and *SG7-2* that we see today in anopheline species of the three different subgenera *Cellia*, *Anopheles* and *Nyssorhynchus. SG7-2* underwent a more recent duplication within the *An. gambiae* species complex, most likely in the last 1.5–2.0 million years. As a consequence, in *An. gambiae* and *An. coluzzii* we found a cluster of three genes arranged in close proximity to each other, whereas in *An. quadriannulatus* and *An. merus* only degenerated copies containing frameshifts could be traced (Fig. 1). As mentioned earlier the mosquito 30 kDa protein possesses antihemostatic properties in virtue of its capacity to bind collagen, a function of crucial importance considering its conservation in both anophelines and culicines. Its duplication, followed by divergence, allowed the C-terminal domain to evolve new binding properties, as displayed by the *An. stephensi* anophensin and by the *An. albimanus* albicin, and provided anopheline mosquitoes with the opportunity to acquire new weapons to fight the inflammatory and hemostatic responses of their hosts.

### Anopheline salivary proteins as markers of host exposure

Salivary proteins injected into vertebrate hosts during blood feeding elicit an anti-saliva humoral response and, as a consequence, individuals repeatedly bitten by arthropods carry circulating antibodies directed against salivary proteins [7, 17]. This anti-saliva antibody response allows for the evaluation of host exposure to a wide range of arthropod disease vectors as diverse as ticks [108], sand flies [109], triatomines [110], tsetse flies [111] and mosquitoes [112–116]. Initial studies in the field were based on the development of immunoassays based on the use of whole saliva, which is difficult to reproducibly collect in large amounts and, being a complex cocktail, may cause problems with potential cross-reactivity. However, sialotranscriptome studies carried out in the last decade revealed both the complexity and the large diversification of blood feeding arthropod salivary repertoires, paving the way to the exploitation of individual recombinant salivary proteins for the development of immunoassays suitable to evaluate host exposure to specific disease vectors. As previously described the sialome of anopheline mosquitoes includes a group of genus-specific salivary proteins that, at least in principle, represent ideal candidates as markers of host exposure to malaria vectors.

So far two *An. gambiae* salivary proteins have been tested as indicators of human exposure to malaria vectors: the gSG6 protein and the antithrombin cE5. The gSG6 protein was the first *An. gambiae* salivary protein shown to be immunogenic to humans [117] and, afterwards, it was validated as marker of human exposure to Afrotropical malaria vectors in different epidemiological settings in Senegal [118–121], Burkina Faso [122, 123], Tanzania [124, 125], Kenya [126] and Uganda [127]. Importantly, the human anti-gSG6 IgG response (i) was short term (i.e. decreased after a few months of absent or low exposure), (ii) it was sensitive enough also in conditions of relatively low vector density and (iii) it was a good marker of exposure to bites of the three major malaria vectors in tropical Africa: *An. gambiae*, *An. arabiensis* and *An. funestus* [128–130]. Moreover, the IgG response to gSG6 proved to be valuable to monitor the efficacy of vector control measures in reducing human-vector contact [131, 132], a precious tool for the implementation and evaluation of malaria control strategies. It should be emphasized that in these studies both the purified recombinant protein and a gSG6-based peptide (gSG6-P1) were employed, indicating that the careful selection of peptides designed on specific salivary proteins can be a reliable and effective strategy, although with the predictable drawback of a loss in marker sensitivity. Among the anophelines analyzed in this study the *An. gambiae* gSG6 shares good degree of identity with orthologues from the main African (*An. arabiensis* 98%, *An. funestus* 80%), Asian (*An. stephensi* and *An. maculatus* 79%, *An. culicifacies* 72%, *An. sinensis* 61%, *An. dirus* 54%) and European (*An. atroparvus* 66%) malaria vectors, whereas a more limited identity (52%) was found with *An. farauti*, which is known as a relevant vector in Solomon Islands, Vanuatu and part of Australia. Considering the fast evolutionary rate of salivary proteins from blood feeding arthropods (see below), and compared to other anopheline salivary proteins analyzed here, this degree of conservation is rather high and may support the additional exploitation of the *An. gambiae* gSG6 as indicator of human exposure to at least Asian anopheline vectors. The “high degree” of conservation found in the anophelines considered in this study is in agreement with the indications of strong purifying selection acting on *gSG6* in an *An. gambiae* population from Burkina Faso [133]. The SG6 protein was not found during previous sialome studies in the *Nyssorhynchus* species *An. albimanus* and *An. darlingi*, the two main malaria vectors in Central and South America, and it was absent in the genome of these species, confirming the initial hypothesis that *SG6* was either acquired from the progenitor of Old World species or lost in the ancestral lineage leading to *Nyssorhynchus*. These observations imply that the IgG response to the gSG6 protein/peptide cannot be a reliable indicator of exposure to anopheline vectors in America, at least not in areas where *An. darlingi* and *An. albimanus* are among the prevalent anopheline vectors. Therefore, serological data as those recently reported by Londono-Renteria and collaborators on the response to the gSG6-P1 peptide in the Americas [134] should be interpreted with caution and taking into consideration these novel anopheline genomic data.

A second *An. gambiae* salivary protein studied to a certain extent for its antigenic properties is the antithrombin polypeptide cE5, which was shown to be more immunogenic than gSG6 [135] and may represent a useful sensitive marker to evaluate efficacy of insecticide impregnated bednets in reducing human-vector contact [102]. Strikingly, the two salivary antigens gSG6 and cE5 evoked a substantially different response in naturally exposed individuals from a malaria hyperendemic area of Burkina Faso with (i) gSG6 inducing a short-lived IgG response, high levels of anti-gSG6 IgG4 antibodies and determining immune tolerance after prolonged exposure, whereas (ii) cE5 evoked a more persistent IgG response, dominated by the IgG1 subclass and not inducing tolerance mechanisms [135]. These observations suggest that beyond their exploitation as exposure markers these two antigens may be useful reagents to study the so far neglected role of *Anopheles* saliva and salivary proteins in the host early immune response to *Plasmodium*.

In order to identify potential additional candidates to be developed as general markers of human exposure to malaria vectors worldwide, we compared the *An. gambiae* genus-specific salivary proteins discussed in the previous section to their orthologues from major malaria vectors from Africa (*An. arabiensis* and *An. funestus*), Asia (*An. stephensi*, *An. culicifacies*, *An. maculatus*, *An. dirus*, *An. sinensis*), Oceania (*An. farauti*), Europe (*An. atroparvus*) and South/Central America (*An. albimanus* and *An. darlingi*) that were included in this study. The salivary proteins hyp4.2, hyp13 and hyp17 were not included in the analysis because only information on species of the series Pyretophorus were available. The SG6 protein, which may also be considered as a sort of internal control, ranked among the best candidates, despite the limitations mentioned above.

Our comparison also allowed to identify the SG7-2 and the hyp6.2 proteins, which are present in both Old World and New World anophelines, as novel potential candidates to be tested in future epidemiological studies as markers of human exposure to malaria vectors worldwide (Table 1). Furthermore, the information reported here (Table 1, Additional file 2, Fig. 1) may also guide studies aimed at identifying species-specific markers, which would allow for example to distinguish human exposure to members of the *An. gambiae* species complex from exposure to *An. funestus* [128].

**Table 1:** Divergence of *An. gambiae* salivary proteins among major malaria vectors

	cE5	**hyp6.2**	hyp8.2	hyp10	hyp12	hyp15	SG2	SG2b	**SG6**	SG7	**SG7-2**
Africa	59–100	**68–96**	39–97	59–99	47–100	58–96	66–95	66–91	**80–98**	68–97	**72–96**
Asia	35–62	**62–72**	21–49	57–67	41–53	42–59	45–74	59–62	**54–79**	55–70	**64–75**
Oceania	54	**64**	44	53	42	nf	55	nf	**52**	60	**67**
Europe	36	**55**	32	abs	abs	59	49	nf	**66**	50	**63**
America South-Cent.	35–37	**42–54**	24–25	abs	abs	43–48	NA	nf	**abs.**	46–47	**47**

The *An. gambiae* genus-specific salivary proteins indicated on the top were compared to orthologues from malaria vectors in the different continents: Africa (*An. arabiensis*, *An. funestus*), Asia (*An. stephensi*, *An. culicifacies*, *An. maculatus*, *An. dirus* and *An. sinensis*), Oceania (*An. farauti*), Europe (*An. atroparvus*), South and Central America (*An. albimanus*, *An. darlingi*). Mature proteins were used for the comparison. Numbers indicate percent of identity or its range when multiple vectors from the same continent were available. Abs, gene not present; nf, gene not found but possibly present or not full-length. The three anopheline-specific salivary proteins more conserved among malaria vectors worldwide are highlighted in bold

### Fast evolutionary rate of mosquito salivary proteins

Interspecific comparison of salivary proteins between anopheline mosquito species has previously shown that salivary proteins diverge at a significantly higher rate than housekeeping ones. This was the case for the pairs *An. gambiae*/*An. stephensi*, *An. gambiae*/*An. funestus* and *An. gambiae/An. darlingi* whose orthologous salivary proteins displayed an average identity of 62, 66 and 53%, respectively, vs the 93, 96 and 86% found for housekeeping proteins [19, 21, 23]. Similar results were also obtained for the culicines *Ae. aegypti/Ae. albopictus* [76]. These observations suggested that salivary genes of mosquitoes (and more generally of blood feeding arthropods) are at an accelerated pace of evolution, perhaps under the selective pressure of the host immune system [6]. Indeed signatures of positive selection were found in a limited set of salivary genes from *An. gambiae* [133]. In addition, over 1000 orthologous genes from species belonging to the subgenus *Cellia* were analyzed for the presence of signatures of positive selection on individual codons. Among the seven different classes analyzed the salivary genes, similarly to immune genes, appeared to have very high rate of positive selected codons [31].

In order to evaluate divergence rates of individual anopheline salivary proteins we used orthologs multiple alignments to perform pairwise species comparisons and calculate the average percent amino acid identity among all anopheline species and among those belonging to the three different subgenera. Only one member of the *An. gambiae* species complex, i.e. *An. gambiae s.s.*, was included in the analysis which, therefore, involved up to 14 different species. Fifty orthologous salivary proteins were considered, due to the restriction to Pyretophorus of SerPro2, hyp4.2 and hyp13. In the subgenus *Cellia* four salivary proteins (hyp37.7-2, SG1a, SG1-like2, hyp8.2) displayed an average identity below 50% and other 23 were in the range of 50 to 70% identity (Additional file 16). Salivary proteins belonging to the category of Enzymes were among the most conserved, with average identity between 73 and 88% excluding epoxy hydrolase. We also selected for comparison 12 housekeeping proteins and found only 2 with percentage of identity below 96%, i.e. Integrin beta 1 (84%) and GSTT1 (glutathione S-transferase theta class 1, 86%). Only two salivary proteins showed identity >90% and comparable to housekeeping: Ag5r3 (95%) and Ag5r6 (94%). However, it should be noted that these two genes, according to Baker and collaborators (2001) and in comparison to other Ag5 family members, are not overexpressed in salivary glands and should not to be considered as typical salivary genes, although we included them in our list (mainly because a low number of *Ag5r3* transcripts were found in a previous transcriptome study and because *Ag5r6* is located close to and originated from *Ag5r3* by gene duplication). Two anopheline salivary proteins, excluding those belonging to the Enzymes category, appeared more conserved as compared to the remaining ones, i.e. gVAG and D7r2 (83 and 82% identity among *Cellia*, respectively): both are members of multigene families and widespread among blood feeding arthropods. The “low” divergence of gVAG and D7r2 among anopheline mosquitoes found here is fully consistent with a previous polymorphism analysis of a few salivary genes in an *An. gambiae* population from Burkina Faso. In this study these genes showed the highest nucleotide diversity values in both coding and non-coding regions and the lowest dN/dS ratios, and they were suggested to be under strong evolutionary constraints negatively selecting replacement substitutions [133]. A situation similar to the one described above for *Cellia* species is found when average identities are calculated including all anophelines.

We also used multiple alignments of orthologous coding sequences to calculate a few additional parameters such as the number of amino acid substitution per site (d), the number of synonimous substitution per synonimous site (dS), the number of nonsynonimous substitution per nonsynonimous site (dN), the ratio dN/dS and the nucleotide diversity per site (Pi) (Additional file 16). Interestingly, members of the SG1 family showed a remarkably high diversity and appeared to be under strong selective pressure. In fact, when the salivary protein genes listed in Additional file 16 were sorted by the dN/dS ratio the seven SG1 family members ranked amongst the thirteen with the higher dN/dS ratio. It is worth pointing out that one of the SG1 family member, the saglin protein, was previously shown to be involved in salivary gland invasion by *Plasmodium* sporozoites [96, 97]. We do not know if other SG1 family members may play similar roles although, as previously mentioned, the SM1 peptide was shown by cross-linking experiments to interact not only with Saglin but also with SG1 [97]. Nevertheless, the very high diversity of saglin and other SG1 family members reported here, which may imply a similarly high inter- and intra-population diversity, raises a question on its possible connection with inter- and intra-specific variation in malaria parasite transmission capacity.

## Conclusions

Exploiting the availability of the genome of sixteen anopheline species [31] we provided here a comprehensive overview of the major salivary protein families of anopheline mosquitoes. Overall, 824 full-length salivary proteins were included in our study, which allowed to identify 79 proteins not previously annotated and to correct 379 wrong predictions. This information was used for multiple alignments, phylogenetic analyses, secondary structure predictions and, importantly, to assemble an hyperlinked excel spreadsheet carrying additional documentation and made available as supplemental material. The anopheline species analyzed here span approximately 100 million years of evolution and the analysis of their salivary repertoires helped shedding some light on the main mechanisms driving the evolution of salivary proteins in anophelines and, more generally, in blood feeding arthropods. Gene duplication, followed by divergence, was one of the major driving forces and certainly a key mechanism, as clearly testified by the several duplicated salivary genes and the large multigene families (i.e. D7, Ag5 and SG1 families). The impact of gene duplication(s) in shaping the anopheline sialomes appears striking when the known *An. gambiae* salivary genes are mapped to their chromosomal location on polytene chromosomes as shown in Additional file 17. This extensive duplication of salivary genes may be not so surprising if one considers the redundant and multifaceted response of vertebrate hosts to tissue injury. Indeed redundant salivary repertoires (with proteins of the same family playing slightly different functions as for the short D7 proteins) may provide an opportunity to more efficiently face host hemostatic and inflammatory responses and, in addition, may contribute to a better adaption to feeding on a panel of different vertebrate hosts. However, gene duplication was not only important in the fine-tuning of salivary functions but also played a role on the evolution of novel salivary protein families and, therefore, of novel functions. This is nicely shown by the SG7 family which provides a paradigm of how salivary proteins with novel binding properties (inhibitors of complement and plasma contact system) evolved from the *30 kDa* gene, encoding a platelet inhibitor binding collagen. Evolutionary pressure also must have played a relevant role and resulted in the very high rate of divergence of salivary proteins among anopheline mosquitoes: these observations further corroborate the idea that salivary genes from blood feeding arthropods should be added to the small number of gene classes under positive selection [31, 133]. Overall, we expect that this extended anopheline sialome catalogue will be useful to the vector biology community, both to clarify the role of the many “orphan” mosquito salivary proteins still without a function and to guide their possible exploitation for epidemiological and vector-host-pathogen interaction studies.

## Methods

### Anopheles species

The following Anopheles species and genome assemblies were used for the identification of orthologues of the 53 *An. gambiae* salivary proteins considered in this study: *Anopheles gambiae* (strain PEST, assembly AgamP4, gene set AgamP4.3), *Anopheles coluzzii* (strain Mali-NIH, assembly AcolM1, gene set AcolM1.2), *Anopheles arabiensis* (strain Dongola, assembly AaraD1, gene set AaraD1.3), *Anopheles quadriannulatus A* (strain SANGQUA, assembly AquaS1, gene set AquaS1.3), *Anopheles merus* (strain MAF, assembly AmerM2, gene set AmerM2.1), *Anopheles melas* (strain CM1001059_A, assembly AmelC2, gene set AmelC2.1), *An christyi* (strain ACHKN1017, assembly AchrA1, gene set AchrA1.3), *Anopheles epiroticus* (strain Epiroticus2, assembly AepiE1, gene set AepiE1.3), *Anopheles funestus* (strain FUMOZ, assembly AfunF1, gene set AfunF1.3), *Anopheles minimus* (strain MINIMUS1, assembly AminM1, gene set AminM1.3), *Anopheles culicifacies* (strain A-37, assembly AculA1, gene set AculA1.3), *Anopheles stephensi* (strain SDA-500, assembly AsteS1, gene set AsteS1.3), *Anopheles maculatus* (strain maculatus3, assembly AmacM1, gene set AmacM1.3), *Anopheles farauti* (strain FAR1, assembly AfarF2, gene set AfarF2.1), *Anopheles dirus spA* (strain WRAIR2, assembly AdirW1, gene set AdirW1.3), *Anopheles atroparvus* (strain EBRO, assembly AatrE1, gene set AatrE1.3), *Anopheles sinensis* (strain SINENSIS, assembly AsinS2, gene set AsinS2.1), *Anopheles albimanus* (strain STECLA, assembly AalbS1, gene set AalbS1.3), *Anopheles darlingi* (strain Coari, assembly AdarC3, gene set AdarC3.3).

### Gene annotation

Protein sequences of the 53 *An. gambiae* salivary proteins listed in Additional file 1 were used to search the genomes of the eighteen anopheline species reported above using the blast tool [136] at the VectorBase web site [32]. Orthologous genes were retrieved through the genome browser and manually annotated using the Artemis tool [33, 137]. The *An. funestus D7L2* and a few salivary genes from *An. darlingi* could not be found searching the genomes of these species but were previously reported among the genes identified through transcriptome analysis [19, 21] and were therefore included in Fig. 1 and marked in red. Fasta files including nucleotide coding sequences (from ATG to stop codon) and amino acid sequences of the 824 full-length anopheline salivary genes analyzed here were compiled and used for mapping to the hyperlinked spreadsheet. Among the 824 full-length sequences, 79 were not previously annotated and represent therefore novel protein coding sequences, whereas 379 represented wrong predictions. These sequences were sent to VectorBase curator for inclusion/correction in the next releases. Fasta files including the nucleotide coding sequences and corresponding polypeptides are included as supplemental material (Additional files 18 and 19).

### Hyperlinked spreadsheet

Protein and their coding sequences were mapped to a hyperlinked spreadsheet as detailed previously [138], including the presence of signal peptide indicative of secretion [139], transmembrane domains [140], matches to the gene ontology database [141], to the NCBI set of proteins from Diptera, as well as rpsblast [142], matches to the Conserved Domains Database [143] and related motif databases. The data was also clustered as described in [77], facilitating organization of the database by sorting on clusters of related proteins.

### Multiple alignments, secondary structure predictions and phylogenetic analysis

Multiple alignments were obtained by Clustal Omega [144] using as input, whenever possible, mature peptides as predicted by SignalP 4.0 [145]. Secondary structure predictions were performed using the PSIPRED server [146]. Alignments obtained by Clustal Omega were imported in MEGA 6 and used for evolutionary analyses [147]. Trees were constructed using the Neighbor-Joining method [148] and tested by 10000 bootstrap replications [149]. The evolutionary distances were computed using the Poisson correction method [150] and are in the units of the number of aminoacid substitutions per site. The pairwise deletion option was used to deal with gaps/missing data. Data used for the construction of phylogenetic trees are provided in Additional file 20.

### Divergence of salivary proteins in anophelines

The diversity of salivary proteins among anophelines was evaluated for 50 salivary proteins (SerPro2, hyp4.2 and hyp13 were excluded because restricted to Pyretophorus species) in up to 14 different species (only *An. gambiae* was included as representative of the complex) using, whenever possible, the sequence of mature proteins. As a control the diversity among orthologues of 12 housekeeping proteins in the different species was also calculated (see Additional file 16). For each protein the mean percent identity among anophelines, *Cellia*, *Anopheles* and *Nyssorhynchus* was calculated as the average of the different pairwise comparisons of the orthologues aligned by Clustal Omega. The number of aminoacid substitution per site (d) was estimated by MEGA 6 (1000 bootstrap replications, Poisson model, uniform rates among sites, pairwise deletion option) [147]. Multiple alignments of the nucleotide coding sequences were constructed using the corresponding aligned aminoacid sequences as scaffold by the RevTrans 1.4 Server [151]. The number of synonimous substitution per synonimous site (dS) and the number of nonsynonimous substitution per nonsynonimous site (dN) were computed by MEGA 6 (1000 bootstrap replications, Nei-Gojobori method, pairwise deletion option). The number of variable sites (S) and the nucleotide diversity per site (Pi) were calculated using DnaSP 5.10 [152].

## Additional files

Additional file 1: List of *An. gambiae* salivary proteins used to search anopheline genomes. Protein names, VectorBase accession numbers, length, predicted molecular weight, location on the *An. gambiae* chromosomes and orientation (F, forward; R, reverse) are reported. Chromosomal locations in colours point to genes arranged in clusters. (PDF 42 kb)
Additional file 2: Hyperlinked spreadsheet carrying 824 full-length salivary proteins from 19 anopheline species. Nucleotide coding sequences, peptide sequences, accession numbers (if available), status (novel or annotation needing correction) and several additional informations (protein length, molecular weight, pI, presence of secretory signal and potential glycosilation sites, results of blast searches, etc.) are reported. (XLSX 938 kb)
Additional file 3: Phylogram of the anopheline apyrase and 5'-nucleotidase proteins. The numbers in the phylogram nodes show the percent bootstrap support for the phylogeny (≥70%). The bar at the bottom indicates 5% aminoacid divergence in the sequences. Species belonging to the subgenera *Cellia*, *Anopheles* and *Nyssorhynchus* are labelled with dots, triangles and diamonds, respectively. Within *Cellia* species belonging to the series Pyretophorus (black), Myzomyia (blue), Neocellia (pink) and Neomyzomyia (yellow) are shown. (PDF 24 kb)
Additional file 4: Cluster of 5 peroxidase genes *An. albimanus.* (Top) Five peroxidase genes are clustered in a region of approximately 15 kb in *An. albimanus*. Sal_Perox is the heme peroxidase with catechol oxidase/peroxidase activity characterized by Ribeiro JM and Valenzuela J (1999) [54]. Sal_Perox2 is the orthologue of the *An. gambiae* AGAP010735 identified during a sialotranscriptome analysis [18] and it is indicated as a *bona fide* salivary peroxidase. The other genes of the cluster are indicated simply as Perox genes due to the absence of any evidence of expression in the salivary glands. (Bottom) Percentage of identity among the different putative proteins as indicated. Note the high identity af anoalb_Perox4 to anoalb_Sal_Perox (72.33%), which is suggestive of a relatively more recent gene duplication. (PDF 1284 kb)
Additional file 5: Alignment and phylogram of anopheline salivary serine proteases Sal_SerPro1-3. (A) Multiple alignment of mature anopheline salivary serine proteases Sal_SerPro1-3. Conserved cysteines (red), fully conserved residues (yellow) and the catalytic triad H, D, S (orange) are highlighted. Residues identical in at least 75% of the aligned sequences are shown in green. Species names are abbreviated with the first three letters of the generic name and the first three-four letters of the specific name. VectorBase accession numbers follow (when available). (B) Phylogram of the anopheline Sal_SerPro 1–3 proteins. The numbers in the phylogram nodes indicate the percent bootstrap support (≥70%) for the phylogeny. The bar at the bottom indicates 5% aminoacid divergence in the sequences. The clades including Sal_SerPro1, Sal_SerPro2 and Sal_SerPro3 are shown with different colours. (PDF 1739 kb)
Additional file 6: Alignment of the anopheline Sal_trypXII. Multiple alignment of the anopheline mature salivary serine protease Sal_trypXII. Conserved cysteines (red), fully conserved residues (yellow) and the catalytic triad H, D, S (orange) are highlighted. The conserved (K/R)GD motif is boxed. Residues identical in at least 75% of the aligned sequences are shown in green. Species names are abbreviated as in Additional file 5 and followed by VectorBase accession numbers (when available). (PDF 74 kb)
Additional file 7: Alignment of the anopheline Antigen 5 family proteins. Multiple alignment of the anopheline mature Ag5 family members showing the fully conserved residues (yellow) and the cysteines (red). The DPGR/K tetrapeptide is boxed with the DPGR highlighted in pink. Species names are abbreviated as in Additional file 5 and followed by VectorBase accession numbers (when available). (PDF 181 kb)
Additional file 8: Phylogram of the anopheline Antigen 5 family proteins. The numbers in the phylogram nodes show the percent bootstrap support for the phylogeny (≥70%). The bar at the bottom indicates 10% aminoacid divergence in the sequences. The four clades including gVAG, Ag5r4 and the two pairs of duplicated genes Ag5r2/Ag5r5 and Ag5r3/Ag5r6 are marked. Dots were used to label anopheline Ag5r2 (red), Ag5r5 (black), Ag5r3 (green) and Ag5r6 (light blue). (PDF 115 kb)
Additional file 9: Alignment of the anopheline 30 kDa family members. Multiple alignment of the 30 kDa proteins from 19 anopheline species. Cysteins are highlighted in red, other fully conserved residues in yellow. Gly, Glu and Asp residues are shown in a dark blackground. The first two blocks represent the acidic N-terminal domain. The two alpha helices deduced from the X-ray-resolved crystal of the *An. stephensi* aapp in complex with a mouse Fab antibody are shown above the alignment as blue cilinders (http://www.rcsb.org/pdb/explore.do?structureId=4okv; [94]). Species names are abbreviated as in Additional file 5 and followed by VectorBase accession numbers (when available). (PDF 95 kb)
Additional file 10: The anopheline SG1 protein family. (A) Schematic representation of the cluster including *SG1*, *SG1a*, *Saglin*, *SG1-like2* and *SG1-like3* on the *An. gambiae* X chromosome. The genes with the direction of transcription, accession numbers, length in nucleotide of coding regions and intervening sequences and the names of the encoded proteins are shown. (B) Phylogram including the 119 full-length SG1 family proteins from anophelines (Additional file 2) plus the *An. darlingi* Saglin and SG1-like3 from a previous transcriptome [21]. The numbers in the phylogram nodes indicate percent bootstrap support for the phylogeny (≥90%). The bar indicates 20% aminoacid divergence in the sequences. The different clades and corresponding family members are colour-coded as follows: SG1 (red), SG1a (green), Saglin (blue), SG1-like2 (pink), SG1-like3 (light blue), SG1b (yellow), TRIO (orange). (PDF 2039 kb)
Additional file 11: Alignment of mosquito 55.3 kDa/56.5 kDa proteins. Multiple alignment of the 16 full-length anopheline 55.3 kDa family members and the three 56.5 kDa proteins from *Cx. pipiens quinquefasciatus*, *Ae. aegypti* and *Ae. albopictus*. Cysteins are highlighted in red and other fully conserved residues in yellow. Residues conserved in at least seventeen of the nineteen aligned sequences (~90) are highlighted in green. The Highly conserved block of 27 amino acids toward the N-terminus is boxed. Species names are abbreviated with the first three letters of the generic name and the first three-four letters of the specific name. Species names are abbreviated as in Additional file 5 and followed by VectorBase accession numbers (when available). (PDF 121 kb)
Additional file 12: Alignment of the anopheline mature cE5/anophelin family members. Residues conserved in at least 2/3 of the sequences (green) or fully conserved (yellow) are highlighted. The amino-terminal conserved region, the DPGR tetrapeptide involved in trombin binding and the RGD tripeptide are boxed. Species names are abbreviated as in Additional file 5 and followed by VectorBase accession numbers (when available). (PDF 1384 kb)
Additional file 13: Alignment and phylogram of the anopheline hyp10/hyp12 proteins. (A) Multiple alignment of the anopheline mature hyp10/hyp12 family members. Fully conserved residues (yellow), cysteins (red) and residues conserved in at least 2/3 of the aligned sequences (green) are highlighted. The predicted alpha helical regions are shown above the alignment as blue cilinders. Species names are abbreviated as in Additional file 5. (B) Phylogram including the 28 full-length hyp10 and hyp12 proteins from *Cellia* species. The numbers in the phylogram nodes indicate percent bootstrap support for the phylogeny (≥70%). The bar indicates 10% aminoacid divergence. Hyp10 and hyp12 family members are labelled by light blue and purple dots, respectively. (PDF 4131 kb)
Additional file 14: Alignment of the anopheline SG6 family proteins. Fully conserved residues (yellow), cysteins (red) and residues conserved in at least 2/3 of the aligned sequences (green) are highlighted. Species names are abbreviated as in Additional file 5. (PDF 48 kb)
Additional file 15: Anopheline SG7 family proteins: alignment, phylogram and comparison to the 30 kDa protein. (A) Multiple alignment of the 37 full-length anopheline SG7 family proteins. Fully conserved residues (yellow), cysteins (red) and residues conserved in at least 2/3 of the aligned sequences (green) are highlighted. Fully conserved residues in SG7 and SG7-2 proteins are also highlited in pink and light blue, respectively. Species names are abbreviated as in Additional file 5. (B) Phylogram of the SG7 anopheline proteins. The numbers in the phylogram nodes indicate percent bootstrap support for the phylogeny (≥70%). The bar indicates 10% aminoacid divergence. SG7, SG7-2 and SG7-3 proteins are labelled by orange triangles and by green and red dots, respectively. (C) Alignment of the *An. gambiae* 30 kDa and SG7 proteins. The different exons are marked with different colours to show exon junction conservation. Cysteins are highlighted in red and signal peptides are boxed. (PDF 1742 kb)
Additional file 16: Divergence of orthologous salivary proteins among anophelines and in the subgenera *Cellia*, *Anopheles* and *Nyssorhynchus*. (Worksheet 1_salivary genes divergence) Protein name, corresponding *An. gambiae* accession number, size in amino acids, location on the *An. gambiae* chromosomes and average percent aminoacid identity ± standard deviation are reported. N, number of sequences; S, number of variable sites; d, number of amino acid substitution per site (average over all sequence pairs); dS, number of synonimous substitutions per synonimous site; dN, number of nonsynonimous substitutions per nonsynonimous site; Pi, nucleotide diversity per site; SE, standard error; SD, standard deviation. Average identities ± standard deviations were calculated for each protein from the pairwise comparisons between the different species. Only *An. gambiae s.s.* was included in the analysis as representative of the *An. gambiae* species complex. The grey shading indicates absence of the gene in the corresponding subgenus. NA, not applicable. Salivary proteins with percentage of identity below 50% (orange), between 50 and 60% (green), 60 and 70% (yellow) and above 90% (light blue) are highlighted. Identities in 12 orthologous housekeeping proteins are shown for comparison. EIF1A, Eukaryotic Initiation Factor 1A; G6PD, glucose-6-phosphate 1-dehydrogenase; GAPDH, glyceraldehyde 3-phosphate dehydrogenase; GSTT1, glutathione S-transferase theta class 1; rpL5, ribosomal protein L5; rpS7, ribosomal protein S7; SDH, succinate dehydrogenase. (Worksheet 2_sorted by dN/dS) Salivary protein genes were sorted by dN/dS ratio. The thirteen with the higher dN/dS ratio and including all the seven SG1 family members are boxed. (XLSX 94 kb)
Additional file 17: A polytene chromosome map of the *An. gambiae* salivary genes. *Anopheles gambiae* salivary genes mapped to their chromosomal location on the polytene chromosomes. Gene duplications are shown in bold and red; unrelated salivary genes located in the same chromosomal division are marked in bold and green. The twenty six additional genes previously identified [18] and not included in this study and their accession ID (when available) are: hyp1.2, AGAP005764, hypothetical 1.2 secreted peptide; hyp3.5, AGAP004836, hypothetical 3.5 putative secreted salivary peptide; hyp5.6, hypothetical 5.6 salivary basic secreted peptide; hyp6.3, AGAP007195, hypothetical 6.3 salivary protein; hyp11, AGAP001713, hypothetical salivary protein 11; hyp14.5, AGAP004883, hypothetical 14.5 similar to Culex 14.5 kDa salivary peptide; hyp14.5-1, AGAP001174, hypothetical 14.5-1 similar to Culex 14.5 kDa salivary peptide; hyp14.6, AGAP002085, hypothetical 14.6 putative secreted protein conserved in insects; hyp36, AGAP000911, hypothetical 36 kDa secreted peptide; C-rich, AGAP011183, cystein-rich repeat containing protein; mucin-like, AGAP002771, mucin-like protein; peroxinectin, peroxinectin precursor; Sal_C Lectin, AGAP006267, salivary c-type lectin; Sal_Calreticulin, AGAP004212, salivary calreticulin; Sal_Chymotryp, salivary chymotrypsin; Sal_cut, AGAP008450, salivary secreted protein – possible cuticle or duct protein; Sal_Galectin, AGAP001197, salivary galectin; Sal_Lyso1, AGAP007347, salivary lysozyme precursor – less abundant form; Sal_Lyso2, AGAP007385, salivary lysozyme precursor – abundant form; Sal-MMP1, matrix metalloproteinase 1 partial – may be secreted; Sal_PPOA1, AGAP004639, secreted serine protease possibly involved with prophenoloxidase activation; Sal_PPOA2, AGAP010968, secreted serine protease possibly involved with prophenoloxidase activation; Sal_ret, AGAP006148, putative secreted salivary protein similar to Drosophila retinin; Selenoprotein, AGAP004986, salivary selenoprotein; SG3, AGAP006507, SG3 protein – salivary mucin; SG10, AGAP003841, gSG10. Figure modified from Supplemental Fig. 1, Coluzzi M. et al., Science 298:1415 (2002) with author’s permission (V. Petrarca). Reprinted with permission from AAAS. (PDF 2400 kb)
Additional file 18: Coding sequences in fasta format. This file includes the coding sequences of the 824 anopheline salivary proteins analyzed in this study and included in Additional file 2 (excel spreadsheet). The file is in fasta format (.fas) and can be viewed with a text editor. (FAS 736 kb)
Additional file 19: Peptide sequences in fasta format. This file includes the amino acid sequences of the 824 anopheline salivary proteins analyzed in this study and included in Additional file 2 (excel spreadsheet). The file is in fasta format (.fas) and can be viewed with a text editor. (FAS 266 kb)
Additional file 20: Phylogenetic data. The zipped file includes data used for the phylogenetic trees reported in Fig. 3 and Additional files 3, 5, 8, 10, 13 and 15. The.meg files are the protein alignments used for the construction of the phylogenetic trees (.mts files). All the files are in MEGA format and can be viewed using the software MEGA (Molecular Evolutionary Genetic Analysis). (ZIP 147 kb)

## Abbreviations

5HT: Serotonin or 5-hydroxytryptamine
ADP: Adenosine diphosphate
Ag5: Antigen 5
AMP: Adenosine monophosphate
ATP: Adenosine triphosphate
CAP: Cystein-rich secretory proteins, antigen 5, pathogenesis-related one proteins
CUB: Complement C1r/C1s, Uegf, Bmp1
CysLT: Cysteinyl leukotrienes
dN: Number of nonsynonimous substitutions per nonsynonimous site
dS: Number of synonimous substitutions per synonimous site
E: Epinephrine or adrenalin
FSG: Female salivary glands
FXII: Factor XII
GPI: Glycophosphatidylinositol
H: Histamine
HK: High molecular weight kininogen
IgG: Immunoglobulin G
MSG: Male salivary glands
NE: Norepinephrine or noradrenalin
OBP: Odorant binding proteins
pI: Isoelectric point
Pi: Nucleotide diversity per site
PSI-BLAST: Position specific iterative basic local alignment search tool
SDS-PAGE: Sodium dodecyl sulphate-polyacrylamide gel electrophoresis
TXA2: Thromboxane A2
vWF: Von Willebrand factor
