# Supplementary material for: Anopheline salivary protein genes and gene families: an evolutionary overview after the whole genome sequence of sixteen Anopheles species
Source: BMC Genomics. 2017 Feb 13;18:153. doi: 10.1186/s12864-017-3579-8 (PMC5307786; doi:10.1186/s12864-017-3579-8)
Supplement: Additional file 1: — List of An. gambiae salivary proteins used to search anopheline genomes. Protein names, VectorBase accession numbers, length, predicted molecular weight, location on the An. gambiae chromosomes and orientation (F, forward; R, reverse) are reported. Chromosomal locations in colours point to genes arranged in clusters. (PDF 42 kb) [file 12864_2017_3579_MOESM1_ESM.pdf]

Protein	Accession	Length (aa)	MW (kDa)	Chromosome	For or Rev
**Ag5 family**					
gVAG	AGAP006421	260	29.01	2L-24D	F
Ag5r2	AGAP006419	257	28.13	2L-24D	F
Ag5r3	AGAP003354	260	28.69	2R-15A	F
Ag5r4	AGAP006420	267	29.94	2L-24D	F
Ag5r5	AGAP006418	266	29.31	2L-24D	F
Ag5r6	AGAP013192	260	28.68	2R-15A	F
**Apy/5'nuc family**					
Apyrase	AGAP011971	558	61.82	3L-45A	F
5'nucleotidase	AGAP011026	570	63.54	3L-41A	R
**cE5/anophelin**	AGAP008004	103	9.86	3R-29C	R
**D7 family**					
D7r1	AGAP008284	165	18.74	3R-30B	R
D7r2	AGAP008282	168	18.48	3R-30B	R
D7r3	AGAP008283	169	18.65	3R-30B	R
D7r4	AGAP008281	165	19.31	3R-30B	R
D7r5	AGAP008280	166	18.81	3R-30B	R
D7L1	AGAP008278	311	35.59	3R-30B	F
D7L2	AGAP008279	315	36.14	3R-30B	F
D7L3	AGAP028120	291	33.68	3R-30B	F
**epoxy_hydrolase**	AGAP011970	397	45.27	3L-45A	R
**hyp4.2-hyp13**					
hyp4.2	AGAP003473	62	6.77	2R-15C	F
hyp13	AGAP003474	56	6.19	2R-15C	F
**hyp6.2-hyp8.2**					
hyp6.2	AGAP006495	85	9.29	2L-25A	R
hyp8.2	AGAP006494	91	9.86	2L-25A	R
**hyp10/hyp12 family**					
hyp10	AGAP008307	90	10.02	3R-30B	F
hyp12	AGAP008306	92	10.14	3R-30B	F
**hyp15/hyp17 family**					
hyp15	AGAP000152	78	8.02	X-4A	R
hyp17	AGAP000151	77	8.10	X-4A	R
**hyp37.7 family**					
hyp37.7	AGAP001988	267	29.34	2R-10A	F
hyp37.7-2	AGAP001989	257	28.71	2R-10A	F
**Salivary amylase**	AGAP006371	876	94.18	2L-24C	R
**Salivary maltase**	AGAP002102	593	67.30	2R-10C	F
**Salivary Peroxydase**	AGAP010735	590	66.55	3L-39C	R
**Salivary SerPro family**					
SerPro1	AGAP011912	395	42.11	3L-44C	R
SerPro2	AGAP011914	398	43.36	3L-44C	F
SerPro3	AGAP011913	399	43.16	3L-44C	F
**Salivary trypXII**		270	28.67	2R-14E	
**SG1 family**					
SG1	AGAP000612	401	46.14	X-1D	F
SG1a	AGAP000611	415	48.34	X-1D	F
Saglin	AGAP000610	431	49.30	X-1D	F
SG1-like2	AGAP000609	401	46.56	X-1D	F
SG1-like3	AGAP000607	392	44.59	X-1D	R
SG1b	AGAP000548	385	43.64	X-1D	F
TRIO	AGAP001374	391	43.82	2R-8A	F
**SG2 family**					
SG2	AGAP006506	114	11.85	2L-25A	R
SG2b	AGAP006504	173	17.67	2L-25A	R
**SG5**	AGAP004334	332	38.21	2R-19A	F
**SG6**	AGAP000150	115	13.09	X-4A	R
**SG7 family**					
SG7	AGAP008216	145	16.30	3R-30A	R
SG7-2	AGAP008215	141	16.39	3R-30A	R
SG7-3	AGAP013724	144	16.74	3R-30A	R
**SG8**	AGAP010647	224	25.43	3L-39C	R
**SG9**	AGAP013423	393	42.79	2R-17B	F
**30 kDa**	AGAP009974	252	26.90	3R-36B	R
**55.3 kDa**	AGAP005822	513	55.25	2L-23A	F
