# Supplementary figures and images for: Anopheline salivary protein genes and gene families: an evolutionary overview after the whole genome sequence of sixteen Anopheles species
Source: BMC Genomics. 2017 Feb 13;18:153. doi: 10.1186/s12864-017-3579-8 (PMC5307786; doi:10.1186/s12864-017-3579-8)

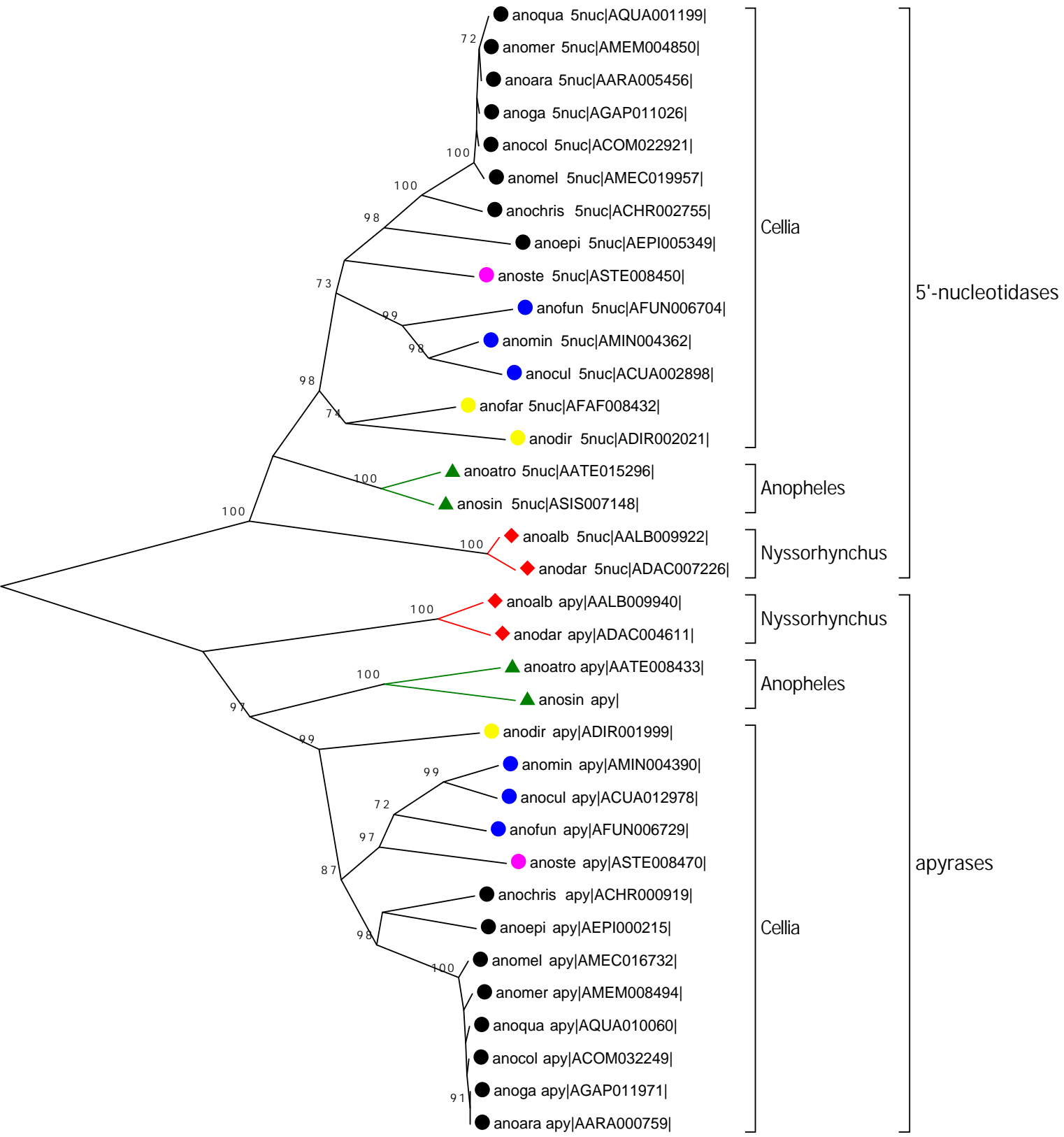

0.05

Supplement: Additional file 3: — Phylogram of the anopheline apyrase and 5'-nucleotidase proteins. The numbers in the phylogram nodes show the percent bootstrap support for the phylogeny (≥70%). The bar at the bottom indicates 5% aminoacid divergence in the sequences. Species belonging to the subgenera Cellia, Anopheles and Nyssorhynchus are labelled with dots, triangles and diamonds, respectively. Within Cellia species belonging to the series Pyretophorus (black), Myzomyia (blue), Neocellia (pink) and Neomyzomyia (yellow) are shown. (PDF 24 kb) [file 12864_2017_3579_MOESM3_ESM.pdf]

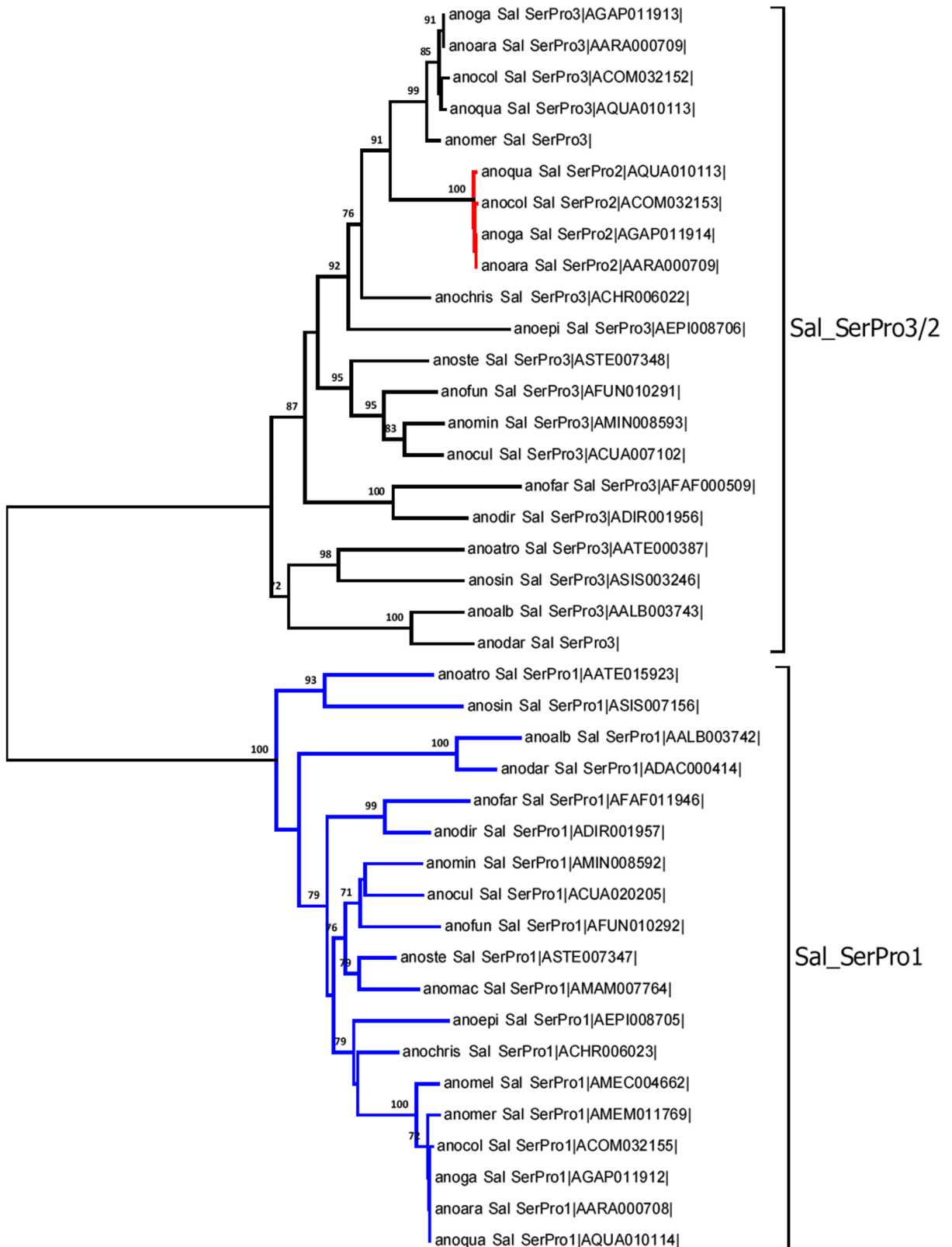

(B)

Supplement: Additional file 5: — Alignment and phylogram of anopheline salivary serine proteases Sal_SerPro1-3. (A) Multiple alignment of mature anopheline salivary serine proteases Sal_SerPro1-3. Conserved cysteines (red), fully conserved residues (yellow) and the catalytic triad H, D, S (orange) are highlighted. Residues identical in at least 75% of the aligned sequences are shown in green. Species names are abbreviated with the first three letters of the generic name and the first three-four letters of the specific name. VectorBase accession numbers follow (when available). (B) Phylogram of the anopheline Sal_SerPro 1–3 proteins. The numbers in the phylogram nodes indicate the percent bootstrap support (≥70%) for the phylogeny. The bar at the bottom indicates 5% aminoacid divergence in the sequences. The clades including Sal_SerPro1, Sal_SerPro2 and Sal_SerPro3 are shown with different colours. (PDF 1739 kb) [file 12864_2017_3579_MOESM5_ESM.pdf]

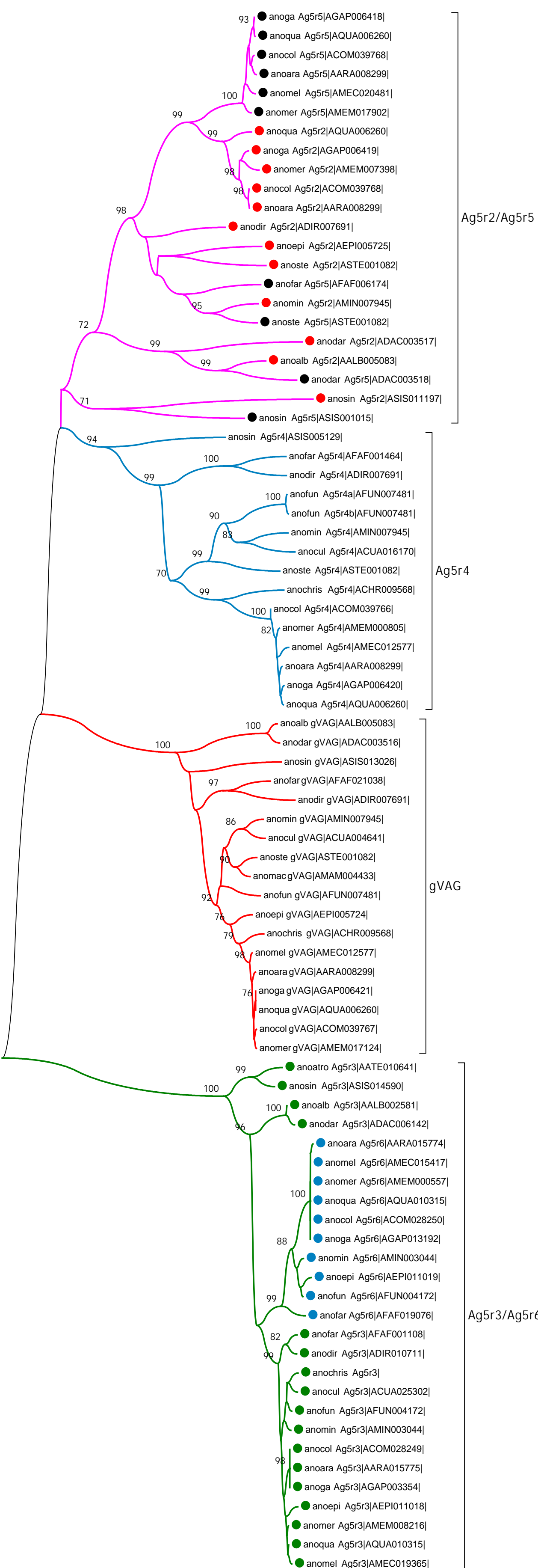

0.1

Supplement: Additional file 8: — Phylogram of the anopheline Antigen 5 family proteins. The numbers in the phylogram nodes show the percent bootstrap support for the phylogeny (≥70%). The bar at the bottom indicates 10% aminoacid divergence in the sequences. The four clades including gVAG, Ag5r4 and the two pairs of duplicated genes Ag5r2/Ag5r5 and Ag5r3/Ag5r6 are marked. Dots were used to label anopheline Ag5r2 (red), Ag5r5 (black), Ag5r3 (green) and Ag5r6 (light blue). (PDF 115 kb) [file 12864_2017_3579_MOESM8_ESM.pdf]

(A)

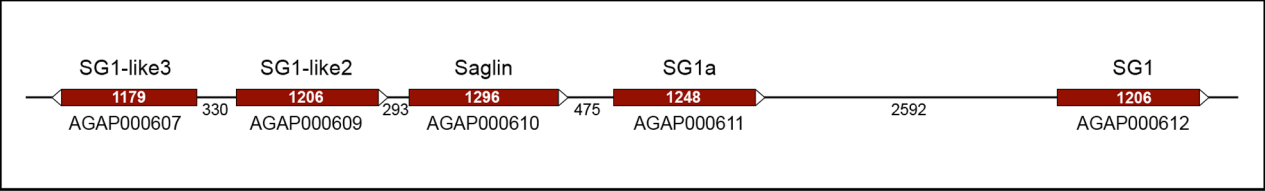

(B)

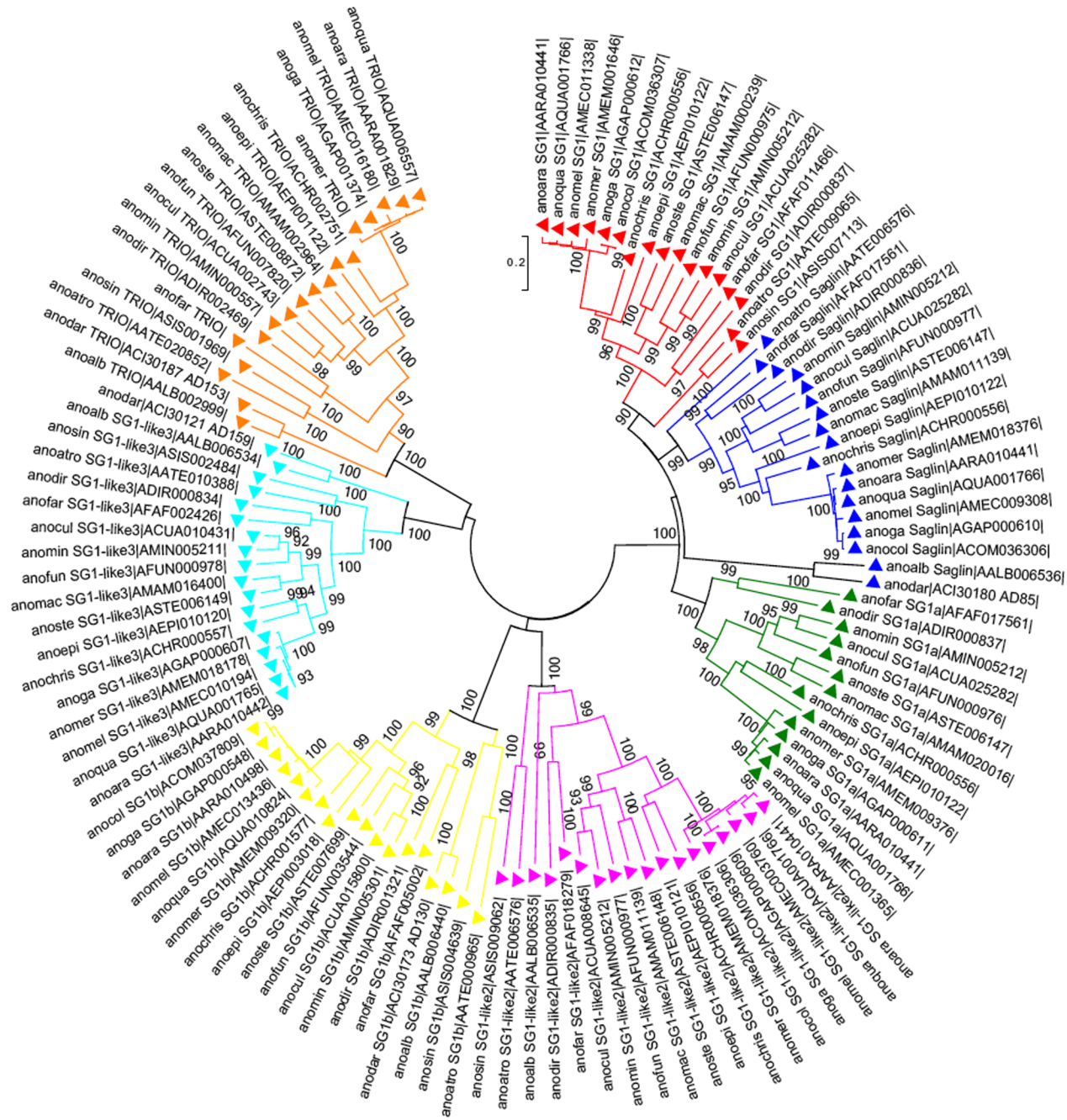

Supplement: Additional file 10: — The anopheline SG1 protein family. (A) Schematic representation of the cluster including SG1, SG1a, Saglin, SG1-like2 and SG1-like3 on the An. gambiae X chromosome. The genes with the direction of transcription, accession numbers, length in nucleotide of coding regions and intervening sequences and the names of the encoded proteins are shown. (B) Phylogram including the 119 full-length SG1 family proteins from anophelines (Additional file 2) plus the An. darlingi Saglin and SG1-like3 from a previous transcriptome [21]. The numbers in the phylogram nodes indicate percent bootstrap support for the phylogeny (≥90%). The bar indicates 20% aminoacid divergence in the sequences. The different clades and corresponding family members are colour-coded as follows: SG1 (red), SG1a (green), Saglin (blue), SG1-like2 (pink), SG1-like3 (light blue), SG1b (yellow), TRIO (orange). (PDF 2039 kb) [file 12864_2017_3579_MOESM10_ESM.pdf]

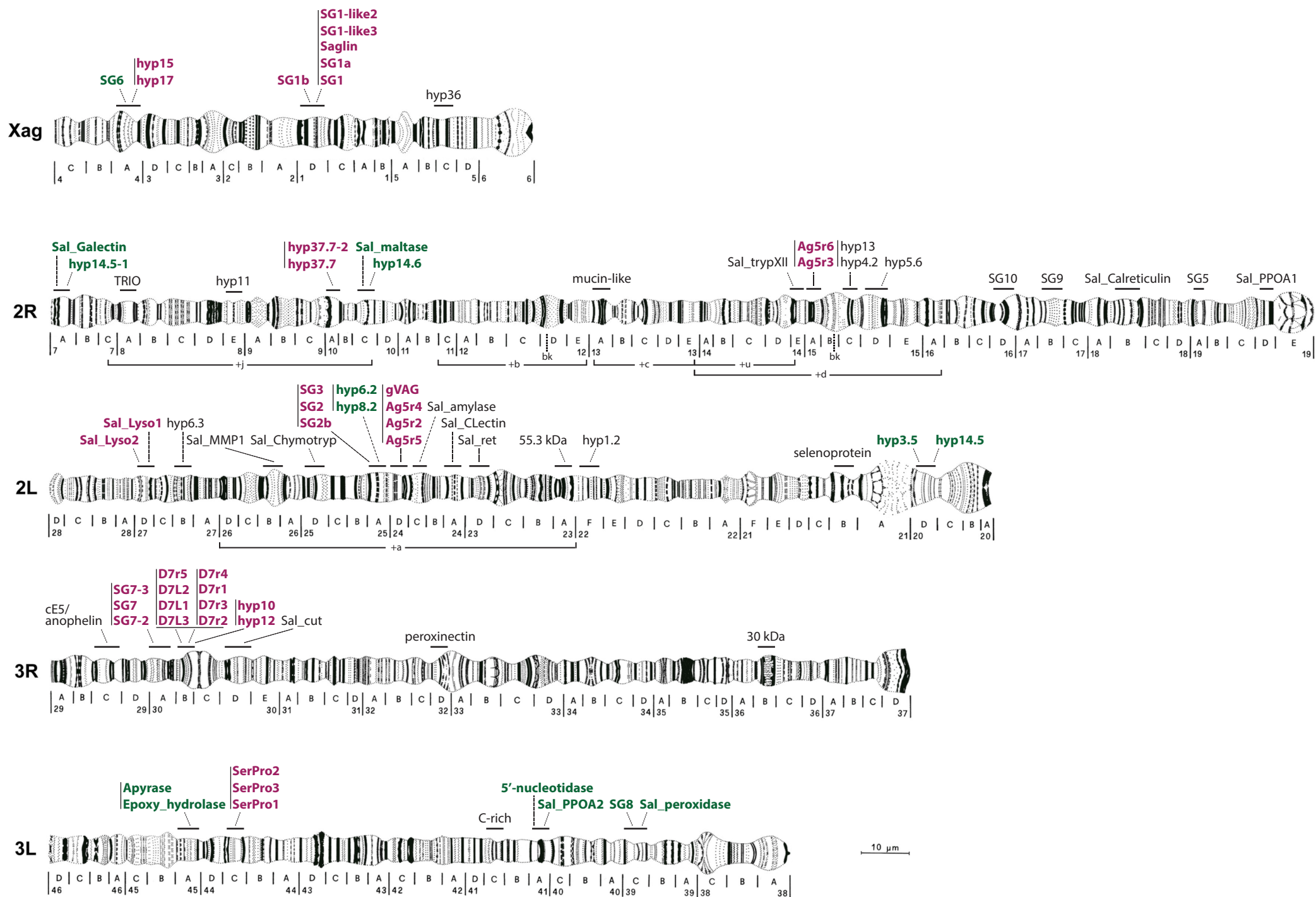

Supplement: Additional file 17: — A polytene chromosome map of the An. gambiae salivary genes. Anopheles gambiae salivary genes mapped to their chromosomal location on the polytene chromosomes. Gene duplications are shown in bold and red; unrelated salivary genes located in the same chromosomal division are marked in bold and green. The twenty six additional genes previously identified [18] and not included in this study and their accession ID (when available) are: hyp1.2, AGAP005764, hypothetical 1.2 secreted peptide; hyp3.5, AGAP004836, hypothetical 3.5 putative secreted salivary peptide; hyp5.6, hypothetical 5.6 salivary basic secreted peptide; hyp6.3, AGAP007195, hypothetical 6.3 salivary protein; hyp11, AGAP001713, hypothetical salivary protein 11; hyp14.5, AGAP004883, hypothetical 14.5 similar to Culex 14.5 kDa salivary peptide; hyp14.5-1, AGAP001174, hypothetical 14.5-1 similar to Culex 14.5 kDa salivary peptide; hyp14.6, AGAP002085, hypothetical 14.6 putative secreted protein conserved in insects; hyp36, AGAP000911, hypothetical 36 kDa secreted peptide; C-rich, AGAP011183, cystein-rich repeat containing protein; mucin-like, AGAP002771, mucin-like protein; peroxinectin, peroxinectin precursor; Sal_C Lectin, AGAP006267, salivary c-type lectin; Sal_Calreticulin, AGAP004212, salivary calreticulin; Sal_Chymotryp, salivary chymotrypsin; Sal_cut, AGAP008450, salivary secreted protein – possible cuticle or duct protein; Sal_Galectin, AGAP001197, salivary galectin; Sal_Lyso1, AGAP007347, salivary lysozyme precursor – less abundant form; Sal_Lyso2, AGAP007385, salivary lysozyme precursor – abundant form; Sal-MMP1, matrix metalloproteinase 1 partial – may be secreted; Sal_PPOA1, AGAP004639, secreted serine protease possibly involved with prophenoloxidase activation; Sal_PPOA2, AGAP010968, secreted serine protease possibly involved with prophenoloxidase activation; Sal_ret, AGAP006148, putative secreted salivary protein similar to Drosophila retinin; Selenoprotein, AGAP004986, salivary selenoprotein; SG [file 12864_2017_3579_MOESM17_ESM.pdf]
