# Supplementary material for: Anopheline salivary protein genes and gene families: an evolutionary overview after the whole genome sequence of sixteen Anopheles species
Source: BMC Genomics. 2017 Feb 13;18:153. doi: 10.1186/s12864-017-3579-8 (PMC5307786; doi:10.1186/s12864-017-3579-8)
Supplement: Additional file 4: — Cluster of 5 peroxidase genes An. albimanus. (Top) Five peroxidase genes are clustered in a region of approximately 15 kb in An. albimanus. Sal_Perox is the heme peroxidase with catechol oxidase/peroxidase activity characterized by Ribeiro JM and Valenzuela J (1999) [54]. Sal_Perox2 is the orthologue of the An. gambiae AGAP010735 identified during a sialotranscriptome analysis [18] and it is indicated as a bona fide salivary peroxidase. The other genes of the cluster are indicated simply as Perox genes due to the absence of any evidence of expression in the salivary glands. (Bottom) Percentage of identity among the different putative proteins as indicated. Note the high identity af anoalb_Perox4 to anoalb_Sal_Perox (72.33%), which is suggestive of a relatively more recent gene duplication. (PDF 1284 kb) [file 12864_2017_3579_MOESM4_ESM.pdf]

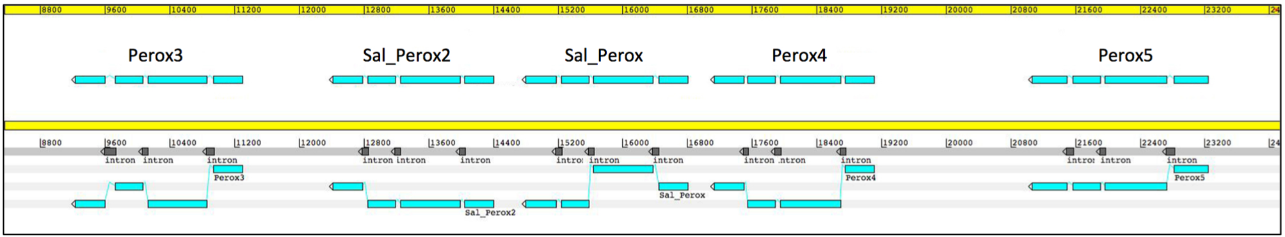

	AGAP010735	Sal_Perox	Sal_Perox2	Perox3	Perox4	Perox5
**AGAP010735**	100	54.44	75.55	54.03	53.74	49.15
**anoalb_Sal_Perox**	54.44	100	53.68	50.6	72.33	47.46
**anoalb_Sal_Perox2**	75.55	53.68	100	52.92	52.81	51.61
**anoalb_Perox3**	54.03	50.6	52.92	100	48.89	45.72
**anoalb_Perox4**	53.74	72.33	52.81	48.89	100	47.8
**anoalb_Perox5**	49.15	47.46	51.61	45.72	47.8	100
