# Supplementary material for: Anopheline salivary protein genes and gene families: an evolutionary overview after the whole genome sequence of sixteen Anopheles species
Source: BMC Genomics. 2017 Feb 13;18:153. doi: 10.1186/s12864-017-3579-8 (PMC5307786; doi:10.1186/s12864-017-3579-8)
Supplement: Additional file 6: — Alignment of the anopheline Sal_trypXII. Multiple alignment of the anopheline mature salivary serine protease Sal_trypXII. Conserved cysteines (red), fully conserved residues (yellow) and the catalytic triad H, D, S (orange) are highlighted. The conserved (K/R)GD motif is boxed. Residues identical in at least 75% of the aligned sequences are shown in green. Species names are abbreviated as in Additional file 5 and followed by VectorBase accession numbers (when available). (PDF 74 kb) [file 12864_2017_3579_MOESM6_ESM.pdf]

```
                                                                                        #
anoga Sal trypXII|                IVRGERL---RKR-DMFPFAVSLQLA-DA----NWTHFCGGTHLGDGWILTAAHCIIPLRKGNLTQMVAVIGGASLDDS-
anocol Sal trypXII|ACOM035823|    IVRGERV---RKR-GMFPFAVSLQLA-DA----NWTHFCGGTHLGDGWILTAAHCIIPLRKGNLTQMVAVIGGASLDDS-
anoara Sal trypXII|AARA001380|    IVRGERV---RQR-GMFPFAVSLQLA-DA----NWTHFCGGTHLGEGWILTAAHCIIPLRKGNLTQMVAVIGGASLDDS-
anoqua Sal trypXII|AQUA003812|    IVRGERV---RKR-GMFPFAVSLQLA-DA----NWTHFCGGTHLGEGWILTAAHCILPLRKGNLTQMVAVIGGASLDDS-
anomer Sal trypXII|AMEM007207|    IVRGERV---RKR-GMFPFAVSLQLA-DA----NWTHFCGGTHLGDGWILTAAHCIIPLRKGNLTQMVAVIGGASLDDS-
anochris Sal trypXII|ACHR003156|  IVRGEPV---RKS-GMFPFAVSLQLA-GA----NWTHFCGGTHLGDGWILTAAHCIIPMQKGNLTQIVAVIGGGILNDN-
anoepi Sal trypXII|AEPI006898|    VVRGEHV---RKR-GMFPFAVSLQLA-DG----NWTHFCGGTHLGEGWILTAAHCILPLQKGNQTQIVAVIGGGNLNDS-
anofun Sal trypXII|AFUN005848|    IVRGETD---RSRKLTFPFAVSLQLA-DD----DRSHFCGGTHLGEGWILTAAHCIISLKKGNLTQIFAQIGGHNLNDS-
anomin Sal trypXII|AMIN000343|    IVRGESD---RGRQLKFPFAVSLQLA-DD----NRTHFCGGTHLGEGWILTAAHCIISVKKGNLTQIFAQIGGVDLSDI-
anocul Sal trypXII|ACUA002290|    IVRGESD---RGRKLMFPFAVSLQLA-DD----SRTHFCGGTHLGDGWILTAAHCIISIKKGNLTQIFAQIGGHDLNDI-
anoste Sal trypXII|ASTE007428|    IVRGEPD---RDGKLMFPFAVSLQLA-DE----RRTHFCGGTHLGDGWILTAAHCIVSVRKGNLTQMFAQIGGHQLNDS-
anofar Sal trypXII|AFAF003068|    IVRGEPI---RQGGDMFPFAVSLQVPTDD----NWKHFCGGTHLGDGWILTAAHCIIPMQKGNLTRIVAQIGGHGLNES-
anodir Sal trypXII|               IVHGESL---R-HRDMFPFAVSLQLA-DD----NWKHFCGGTHLGNGWILTAAHCIIPMQKGNLTRIVAQIGGRSLNDS-
anoatro Sal trypXII|AATE017341|   IVRSESD-GGRPPSALFPFAVSLQLI-DR----AWEHFCGGTHLGDGWILTAAHCVVPFN---HTQIVAQIGGQNLNNG-
anoalb Sal trypXII|AALB006325|    IVRYEVA-DSLSASELFPFAVSLQLVALDGA-KRPQHFCGGTYLGRGWILTAAHCVIAVN---QSNIVAQLGGHELEQEP
anodar Sal trypXII|ADAC004451|    IVRNEVAEGGLTATELFPFAVSLQLVGPDGGTYWPQHFCGGTYLGRGWILTAAHCVIAVN---QSRIVAQLGGHELELRE
                                  :*: *           ********:           ******:** *********:: ..    :.:.* :**  *.

                                                                     #
anoga Sal trypXII|                ------------TVERLPIVETHLLDSYNPVTMVGDIALLRAALPTTPSSKP---EGDT-QSTLRLPNDTYRTAANGELC
anocol Sal trypXII|ACOM035823|    ------------TVERLPIVETHLLDSYNPVTMVGDIALLRAALPTTPSSTP---EGDR-QSMLRLPNDTYRTAANGELC
anoara Sal trypXII|AARA001380|    ------------TVERLPIVETHLLDSYNPVTMVGDIALLRAALPTTPSSKP---EGDT-QSTLRLPNDTYRTAANGELC
anoqua Sal trypXII|AQUA003812|    ------------TVERLPIVETHLLDSYNPVTMVGDIALLRAALPTTPSSKP---ESDP-RSMLRLPNDTYRAAANGELC
anomer Sal trypXII|AMEM007207|    ------------TVERLPIVETHLLDSYNPVTMVGDIALLRAALPTTPSSKL---EDDP-RSMLSLPNDTYRTAANGELC
anochris Sal trypXII|ACHR003156|  ------------TVHRLPIVETHIPDSYNPVTMVGDIALLRAALPTTT-GKP---KTGT-QSTLRLPNESYRSAVNGELC
anoepi Sal trypXII|AEPI006898|    ------------SVQRLPIVETHILSSYNPVTMVGDIALLRAVLPRNSSGTS---GTELQSSILRLPDESYRTAVNGELC
anofun Sal trypXII|AFUN005848|    ------------SADRYPIVETHILRSYNPVTMVGDIAMLRAALPIAHKFQS------DPQAPLRLPDESYHTAINGEQC
anomin Sal trypXII|AMIN000343|    ------------TADRYPIVETHILRSYNPVTMVGDIAVLRASLPTTRTFQS------DPRSPLRLPDESYRAAVNGEQC
anocul Sal trypXII|ACUA002290|    ------------TADRYPIAETHILRSYNPVTMVGDIALLRASLPTVRSFQS------DPWAPLRLPDESYRTAVNGEQC
anoste Sal trypXII|ASTE007428|    ------------TADRFPIVETHLLRSYNPVTMVGDIALLRAELPTGRRFES------DPQTPLRLPDDSK--AANGELC
anofar Sal trypXII|AFAF003068|    ------------TIERYPIVETHILPEYNPVTMAGDIALLRADVVWTQ------------PIRLRLPTESHGTLVNGEMC
anodir Sal trypXII|               ------------TVERYPIVETHILHDYNPVTMVGDIALLRADVVWTE------------PVRLQLPYESYRTPVNGEMC
anoatro Sal trypXII|AATE017341|   ------------TVERYPIVEMQILRTYNPVTMVGDIALLRAALVEVSGRAI--RPRRRDTKALRLPARAEGTVVNGELC
anoalb Sal trypXII|AALB006325|    AAAELPAG----PPHRYPVVEVRILGSYNPVTMVGDIALLRIEAPVPKGGTAAERALWQRRHQLRLPLPTDRIAMNGEMC
anodar Sal trypXII|ADAC004451|    AAVEHPAGSSQPPVARYPVVEVRILANYNPITMVGDIALLRIEAVGTAAKTLWRRRRGQQQEQLRLPLPTDRIAMNGEMC
                                                 * *:.* ::   ***:**.****:**                      * **  :     *** *

                                                                                              #
anoga Sal trypXII|                YIFGYGSGSYDGPISRTLHYGTVLALELDRCIGMMGAVVAPPTDSGMFCAIGRSDACKGDSGGGYVCQRPPS-TQFVLRG
anocol Sal trypXII|ACOM035823|    YIFGYGSGSYDGPISRTLHYGTVLALELDRCIGMMGAVVAPPTDSGMFCAIGRSDACKGDSGGGYVCQRPPS-TQFVLRG
anoara Sal trypXII|AARA001380|    YIFGYGSGSYDGPISRTLHYGTVLALELDRCIGMMGAVVAPPTDSGMFCAIGRSDACKGDSGGGYVCQRPPS-TQFVLRG
anoqua Sal trypXII|AQUA003812|    YIFGYGSGSYDGPISRTLHYGTVLALELDRCIGMMGAVVAPPTDSGMFCAIGRSDACKGDSGGGYVCQRPPS-TQFVLRG
anomer Sal trypXII|AMEM007207|    YIFGYGSGSYDGPISRTLHYGTVLALELDRCIGMMGAVVAPPTDSGMFCAIGRSDACKGDSGGGYVCQRPPS-TQFVLRG
anochris Sal trypXII|ACHR003156|  YIFGYGSESYDGPISRTLHYGTVLALGLDSCIGMMGAVVAPPPDSGMFCAIGRSDACKGDSGGGYVCQRPFS-TQFVLRG
anoepi Sal trypXII|AEPI006898|    YIFGYGSDSYDGPISGTLRYGTVLALELDSCIGMMGAVVAPPPDSGMFCAIGRSDACKGDSGGGYVCQRPHT-TQFVLRG
anofun Sal trypXII|AFUN005848|    YIFGYGSDSYDGPISRSLHYGTVLALDLDSCIGMMGAVVAPPPDSGMFCAIGRSDACKGDSGGGYVCRGPFS-TQFVLRG
anomin Sal trypXII|AMIN000343|    YIFGYGSDSYDGPISRSLHYGTVLALELDSCIGMMGAVVAPPPDSGMFCAIGRSDACKGDSGGGYVCRQPFS-TQFVLRG
anocul Sal trypXII|ACUA002290|    YIFGYGSESYDGPISKSLHYGTVLALELDSCIGMMGAVVAPPPDSGMFCAIGRSDACKGDSGGGYVCRQPFS-TQFVLRG
anoste Sal trypXII|ASTE007428|    YIFGYGSDSYDGPISQTLHYGTVLALDLDSCVGMMGAVVAPPPDSGMFCAIGRSDACKGDSGGGYVCRAPFS-TQFVLRG
anofar Sal trypXII|AFAF003068|    YIFGYGSDSYEGPISGTLRYGTVLALDLNRCVGMMGAVVAPPPDSGMFCAIGRSDACRGDSGGGYVCKKPFS-SRYVLRG
anodir Sal trypXII|               YIFGYGSESYEGPISDTLRYGTVLALDLSRCIGMMGAVVAPPPDSGMFCAIGRSDACKGDSGGGYVCQKPFS-SQYVLRG
anoatro Sal trypXII|AATE017341|   YIFGYGSESYDGPVSETLRYGTVLAFELDSCIGMMGAVVAPPPDSGMFCAIGQADACKGDSGGGYVCRKPSS-SQFVLRG
anoalb Sal trypXII|AALB006325|    YIFGYGSEDYDGPISRTLRYGTVLALEPDRCVGMMGAVVAPPPDSGMFCAIGRADACRGDSGGGYVCRSPSSKGRFVLRG
anodar Sal trypXII|ADAC004451|    YIFGYGSEEYDGPISKSLRYGTVLALEPDRCIGMMGAVVAPPPDSGMFCAIGRADACRGDSGGGYVCRSPSSDGPFVLRG
                                  ******* .*:**:* :*:******:  . *:********** *********::***:*********: * :   :****

anoga Sal trypXII|                VISYGVGCGAPGTPGVYTDVAYYLSHFPIGPTV---------
anocol Sal trypXII|ACOM035823|    VISYGVGCGAPGTPGVYTDVAYYLSHFPIGPTV---------
anoara Sal trypXII|AARA001380|    VISYGVGCGAPGTPGVYTDVAYYLSHFPIGPTV---------
anoqua Sal trypXII|AQUA003812|    VISYGVGCGAPGTPGVYTDVAYYLRHFPIGPTV---------
anomer Sal trypXII|AMEM007207|    VISYGVGCGAPGTPGVYTDVAYYLRHFPIGPTV---------
anochris Sal trypXII|ACHR003156|  VISYGVGCGAPGTPGVYTDVAYYLQQTPISAIIGSA------
anoepi Sal trypXII|AEPI006898|    VISYGVGCGAPGTPGVYTDVGYYRQHYPFGTLIGSS------
anofun Sal trypXII|AFUN005848|    IISYGVGCGAPGTPGVYTDVGYYLQHYPIGTIIGFA------
anomin Sal trypXII|AMIN000343|    IISYGVGCGAPGTPGVYTDVGYYLQHYPIGSIIGFS------
anocul Sal trypXII|ACUA002290|    IISYGVGCGAPGTPGVYTDVGYYLQHYPIGAIIGLA------
anoste Sal trypXII|ASTE007428|    VISYGVGCGATGTPGVYTDVAYYLRNYPIGSLIGTA------
anofar Sal trypXII|AFAF003068|    IISYGVGCGAIGTPGVYTDVGYYLQHSPIGSLIGPTTCLK--
anodir Sal trypXII|               VISYGVGCGAPGTPGVYTDVAYYLQHSPIGSFVGASSPK---
anoatro Sal trypXII|AATE017341|   IISYGVGCGAAGTPGVYTDVAYYLHHYPMSSIKSY-------
anoalb Sal trypXII|AALB006325|    IISYGVGCGAAGTPGVYTDVAYYLHHYQPAITAFPSDRMRSA
anodar Sal trypXII|ADAC004451|    IISYGVGCGAAGTPGVYTDVAYYLHHYQPATTSLLSDRMRFP
                                  :********* *********.**  .   .
```
