# Supplementary material for: Anopheline salivary protein genes and gene families: an evolutionary overview after the whole genome sequence of sixteen Anopheles species
Source: BMC Genomics. 2017 Feb 13;18:153. doi: 10.1186/s12864-017-3579-8 (PMC5307786; doi:10.1186/s12864-017-3579-8)
Supplement: Additional file 9: — Alignment of the anopheline 30 kDa family members. Multiple alignment of the 30 kDa proteins from 19 anopheline species. Cysteins are highlighted in red, other fully conserved residues in yellow. Gly, Glu and Asp residues are shown in a dark blackground. The first two blocks represent the acidic N-terminal domain. The two alpha helices deduced from the X-ray-resolved crystal of the An. stephensi aapp in complex with a mouse Fab antibody are shown above the alignment as blue cilinders (http://www.rcsb.org/pdb/explore.do?structureId=4okv; [94]). Species names are abbreviated as in Additional file 5 and followed by VectorBase accession numbers (when available). (PDF 95 kb) [file 12864_2017_3579_MOESM9_ESM.pdf]

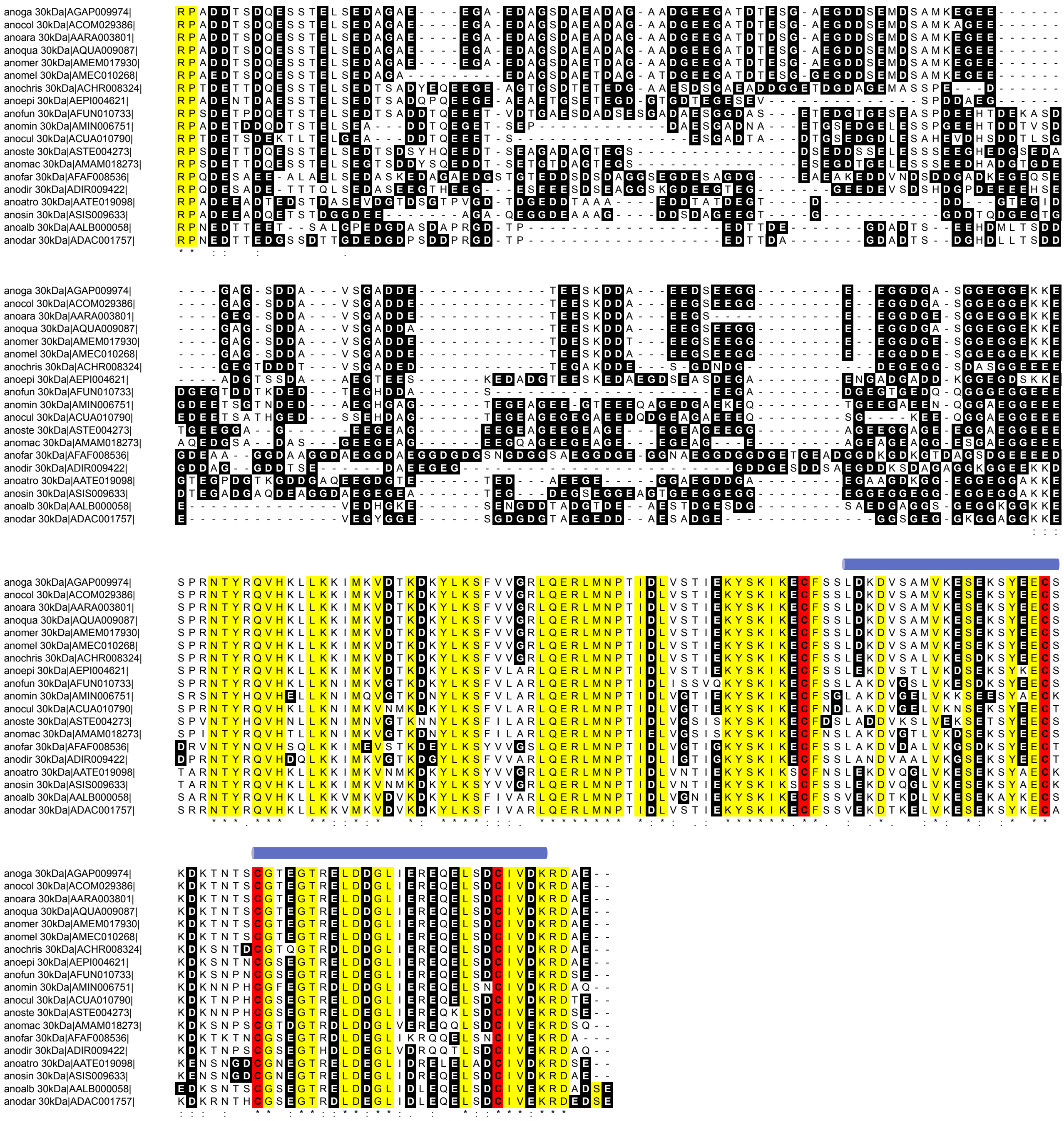
anoga 30kDa|AGAP009974|
anocol 30kDa|ACOM029386|
anoara 30kDa|AARA003801|
anoqua 30kDa|AQUA009087|
anomer 30kDa|AMEM017930|
anomel 30kDa|AMEC010268|
anochris 30kDa|ACHR008324|
anoepi 30kDa|AEPI004621|
anofun 30kDa|AFUN010733|
anomin 30kDa|AMIN006751|
anocul 30kDa|ACUA010790|
anoste 30kDa|ASTE004273|
anomac 30kDa|AMAM018273|
anofar 30kDa|AFAF008536|
anodir 30kDa|ADIR009422|
anoatro 30kDa|AATE019098|
anosin 30kDa|ASIS009633|
anoalb 30kDa|AALB000058|
anodar 30kDa|ADAC001757|
