# Supplementary material for: Anopheline salivary protein genes and gene families: an evolutionary overview after the whole genome sequence of sixteen Anopheles species
Source: BMC Genomics. 2017 Feb 13;18:153. doi: 10.1186/s12864-017-3579-8 (PMC5307786; doi:10.1186/s12864-017-3579-8)
Supplement: Additional file 13: — Alignment and phylogram of the anopheline hyp10/hyp12 proteins. (A) Multiple alignment of the anopheline mature hyp10/hyp12 family members. Fully conserved residues (yellow), cysteins (red) and residues conserved in at least 2/3 of the aligned sequences (green) are highlighted. The predicted alpha helical regions are shown above the alignment as blue cilinders. Species names are abbreviated as in Additional file 5. (B) Phylogram including the 28 full-length hyp10 and hyp12 proteins from Cellia species. The numbers in the phylogram nodes indicate percent bootstrap support for the phylogeny (≥70%). The bar indicates 10% aminoacid divergence. Hyp10 and hyp12 family members are labelled by light blue and purple dots, respectively. (PDF 4131 kb) [file 12864_2017_3579_MOESM13_ESM.pdf]

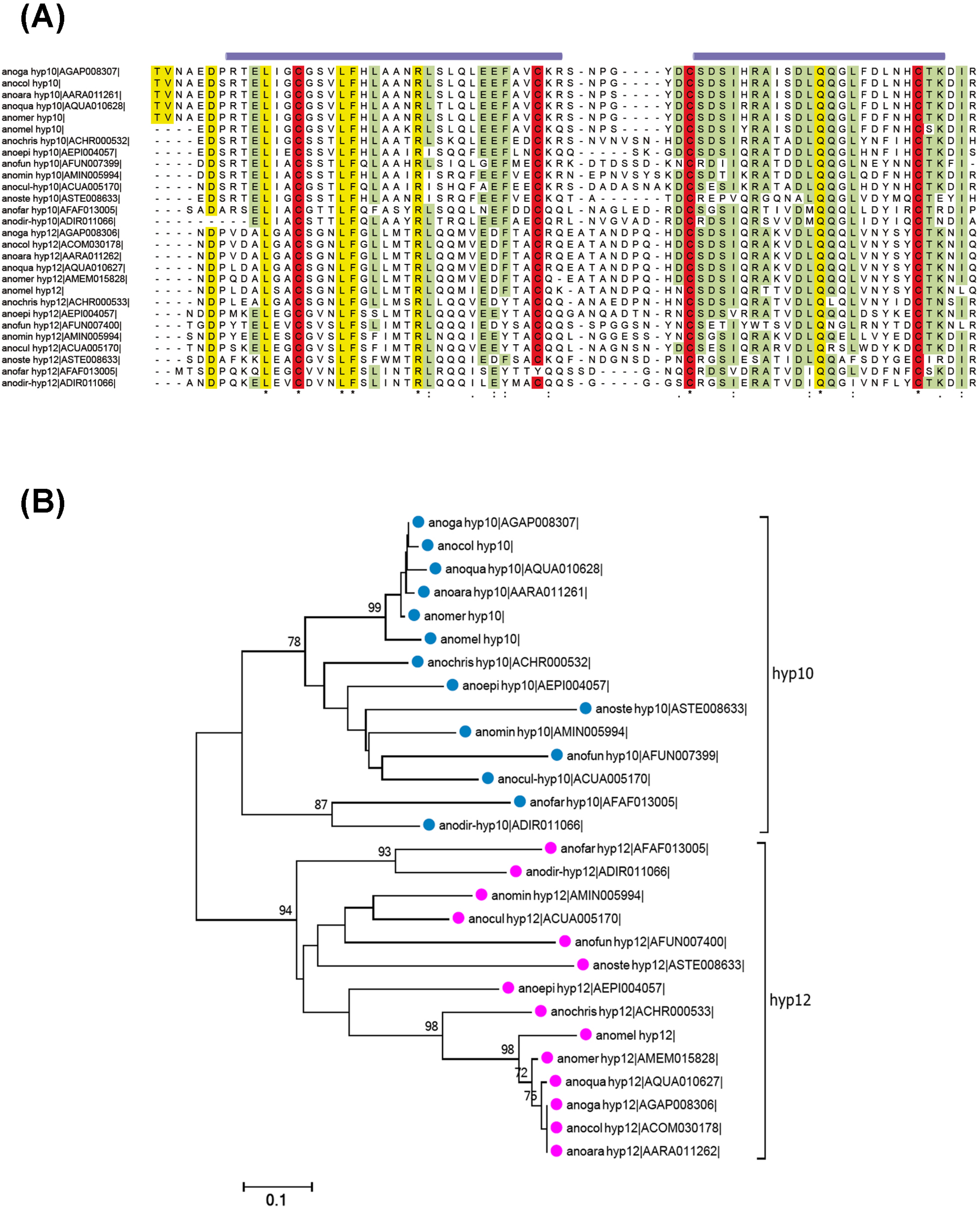
(A)
anoga hyp10|AGAP008307|
anocol hyp10|
anoara hyp10|AARA011261|
anoqua hyp10|AQUA010628|
anomer hyp10|
anomel hyp10|
anochris hyp10|ACHR000532|
anoepi hyp10|AEPI004057|
anofun hyp10|AFUN007399|
anomin hyp10|AMIN005994|
anocul-hyp10|ACUA005170|
anoste hyp10|ASTE008633|
anofar hyp10|AFAF013005|
anodir-hyp10|ADIR011066|
anoga hyp12|AGAP008306|
anocol hyp12|ACOM030178|
anoara hyp12|AARA011262|
anoqua hyp12|AQUA010627|
anomer hyp12|AMEM015828|
anomel hyp12|
anochris hyp12|ACHR000533|
anoepi hyp12|AEPI004057|
anofun hyp12|AFUN007400|
anomin hyp12|AMIN005994|
anocul hyp12|ACUA005170|
anoste hyp12|ASTE008633|
anofar hyp12|AFAF013005|
anodir-hyp12|ADIR011066|
(B)
hyp10
hyp12
99
78
87
93
94
98
98
72
75
0.1
