# Supplementary material for: Anopheline salivary protein genes and gene families: an evolutionary overview after the whole genome sequence of sixteen Anopheles species
Source: BMC Genomics. 2017 Feb 13;18:153. doi: 10.1186/s12864-017-3579-8 (PMC5307786; doi:10.1186/s12864-017-3579-8)
Supplement: Additional file 14: — Alignment of the anopheline SG6 family proteins. Fully conserved residues (yellow), cysteins (red) and residues conserved in at least 2/3 of the aligned sequences (green) are highlighted. Species names are abbreviated as in Additional file 5. (PDF 48 kb) [file 12864_2017_3579_MOESM14_ESM.pdf]

```
anoga SG6|AGAP000150|       ---------EKVWVDRDNVYCGHLDCTRVATFKGERFCTLCDTRHFCECKE
anocol SG6|ACOM040125|      ---------EKVWVDRDKVYCGHLDCTRVATFKGERFCTLCDTRHFCECKE
anoara SG6|AARA001017|      ---------EKVWVDRDKVYCGHLDCTRVATFKGERFCTLCDTRHFCECKE
anoqua SG6|AQUA002523|      ---------EKVWVDRDKVYCGHLDCTRVATFKGERFCTLCDTRHFCECKE
anomer SG6|AMEM011397|      ---------EKVWVDRDNVYCGHLDCTKVATFKGERFCTLCDTRHFCECKE
anomel SG6|AMEC001483|      ---------EKVWVDRDKVYCGHLDCTRVATFKGERFCTLCDTRHFCECKE
anochris SG6|ACHR005518|    ---------EKVWVDRDQVYCGHIDCTKVATFKGERFCSPCDTRHFCECKE
anoepi SG6|AEPI010446|      ---------EKVWVDRDKVYCEHIDCTKVATFKGERFCSPCDTRHFCECKE
anofun SG6|AFUN003883|      -VGGPYATAEKVWVDRDQVYCGHIDCTRVATFKGERFCSPCDTRHFCECKE
anomin SG6|AMIN005447|      ---------EKVWVDRDRVYCGHIDCTRVATYKGERFCSPCDTRHFCECKE
anocul SG6|                 ---------EKVWVDRDKVYCSHIDCRYDATYKGERFCTLCDTQHFCECKE
anoste SG6|ASTE000264|      QTSGPYAAAEKVWVDRDKVYCEHIDCTRVATYKGERFCSPCDTRHFCECKE
anomac SG6|AMAM005167|      QSGGPYATAAKVWVDRDKVYCEHIDCTRLATFKGERFCSPCDTRHFCECKE
anofar SG6|AFAF016250|      ----------DDGIDQDKVFCGHLDCRKIATYKKEKFCNPCDNRHYCECVE
anodir SG6|ADIR000828|      ----------QTWIDRDKTYCEHIDCTKLAKYKGEKFCSPCDTRHYCECKE
anoatro SG6|AATE009364|     ------QEKQKQWIDRDSVYCGHIDCTKLATFKGEKFCSPCDTHHYCECKE
anosin SG6|ASIS003087|      -------EKQKQWIDRDAVYCGHIDCTKLATFKGEKFCSPCDTEHYCECKE
                                      .  :*:* .:* *:**   *.:* *:**. **..*:*** *

anoga SG6|AGAP000150|       TREPLPYMYACPGTEPCQSSDRLGSCSKSMHDVLCDRIDQAFLEQ------
anocol SG6|ACOM040125|      TREPLPYMYACPGTEPCQSSDRLGSCSKSMHDVLCDRIDQAFLEQ------
anoara SG6|AARA001017|      TREPLPYMYACPGTEPCQSSDRLGSCSKTMHDVLCDRIDQAFLEQ------
anoqua SG6|AQUA002523|      TREPLPYMYACPGTEPCQSSDRLGSCSKTMHDVLCDRIDQAFLEQ------
anomer SG6|AMEM011397|      TREPLPYMYACPGTEPCQSSDRLGSCRKTMHDELCNRIDQAFLEQ------
anomel SG6|AMEC001483|      TREPLPYMYACPGTEPCQSSDRLGSCSKSMHDVLCDRIDQAFLEQ------
anochris SG6|ACHR005518|    TLESLPYMYACPGTEPCQTSDRRGSCTKTMHDDLCNLIDKPFLEQ------
anoepi SG6|AEPI010446|      TREPLPYMYACPGTEPCQTSDRRGACTKTMRDELCSLIDKPFLEQ------
anofun SG6|AFUN003883|      TLESLPYMYECPGTEPCQTSDRRGTCQKTMHDELCSLIDKPFLEQ------
anomin SG6|AMIN005447|      TAEPLPYMYECPGSTPCQTSDRRGTCQTTMEDKLCSLIDKPFLEQ------
anocul SG6|                 IKESLPYMYACPGSEPCKTSDRRGNCQKTMDDKLCSQIDKPFLE-------
anoste SG6|ASTE000264|      TKESLPYMYACPGTEQCQTSDRRGSCQQTMSDELCSRIDQAFLEA------
anomac SG6|AMAM005167|      TKESLPYMYACPGMEQCQTSDRRGSCQKTMSDELCNRIDLAFLEQIEDYVI
anofar SG6|AFAF016250|      TSESLPYLRSCSGSTECQTRDRLGRCQKTLSDDKCKLID------------
anodir SG6|ADIR000828|      VRESLPYLGTCPGSGECQKTDSRGKCEKTLHNNLCSLIDKPYM--------
anoatro SG6|AATE009364|     TREPLPYLQACKG-GACQSTDRRGSCSRTMRDDLCSHIDEAFRYL------
anosin SG6|ASIS003087|      TKESLPYMYKCPGSGACQTTDRRGACRRTMDDDLCSHIDEAYRYL------
                              * ***:  * *   *:. *  * *  :: :  *. **
```
