# Supplementary material for: Anopheline salivary protein genes and gene families: an evolutionary overview after the whole genome sequence of sixteen Anopheles species
Source: BMC Genomics. 2017 Feb 13;18:153. doi: 10.1186/s12864-017-3579-8 (PMC5307786; doi:10.1186/s12864-017-3579-8)
Supplement: Additional file 15: — Anopheline SG7 family proteins: alignment, phylogram and comparison to the 30 kDa protein. (A) Multiple alignment of the 37 full-length anopheline SG7 family proteins. Fully conserved residues (yellow), cysteins (red) and residues conserved in at least 2/3 of the aligned sequences (green) are highlighted. Fully conserved residues in SG7 and SG7-2 proteins are also highlited in pink and light blue, respectively. Species names are abbreviated as in Additional file 5. (B) Phylogram of the SG7 anopheline proteins. The numbers in the phylogram nodes indicate percent bootstrap support for the phylogeny (≥70%). The bar indicates 10% aminoacid divergence. SG7, SG7-2 and SG7-3 proteins are labelled by orange triangles and by green and red dots, respectively. (C) Alignment of the An. gambiae 30 kDa and SG7 proteins. The different exons are marked with different colours to show exon junction conservation. Cysteins are highlighted in red and signal peptides are boxed. (PDF 1742 kb) [file 12864_2017_3579_MOESM15_ESM.pdf]

(A)

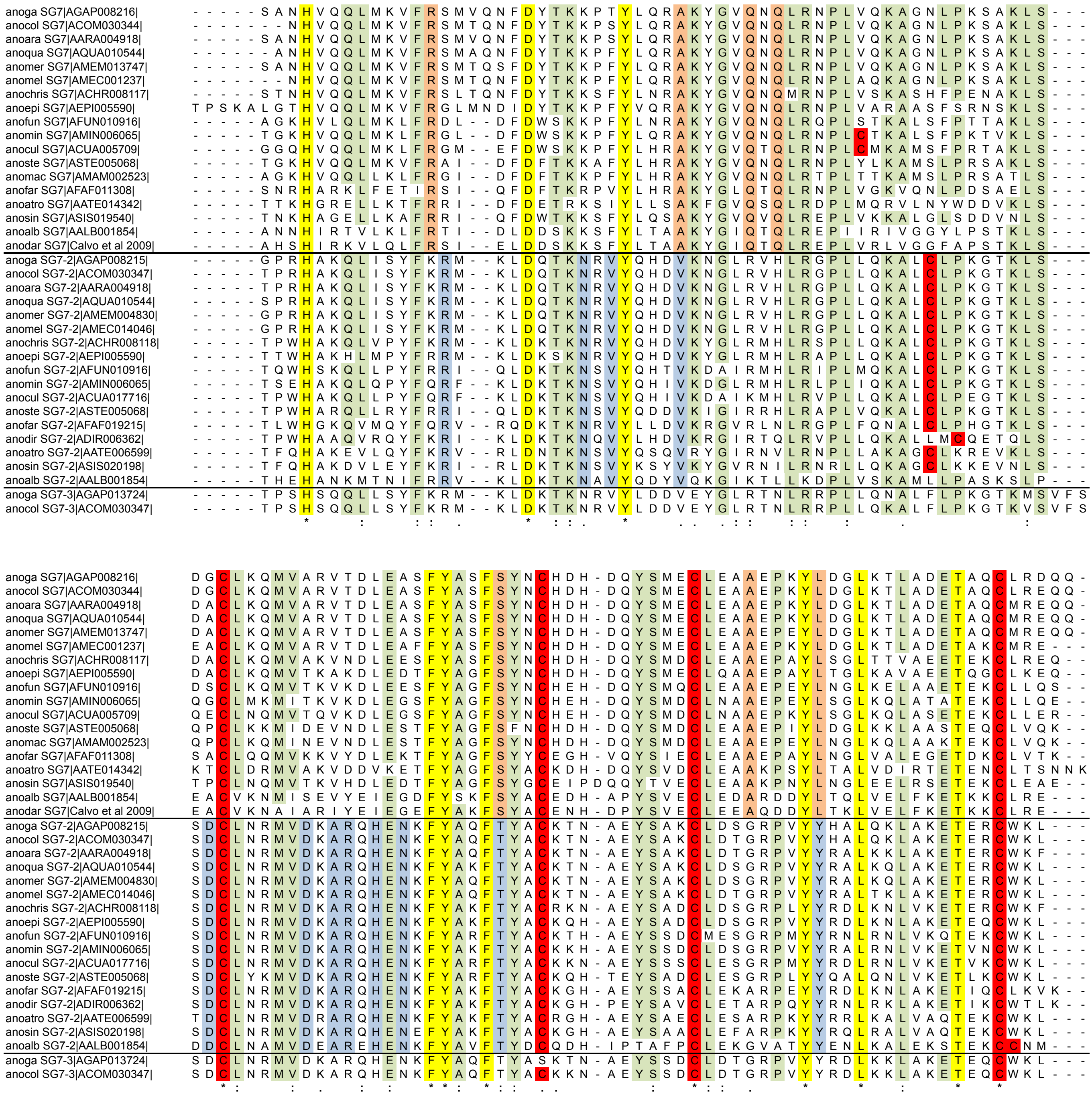

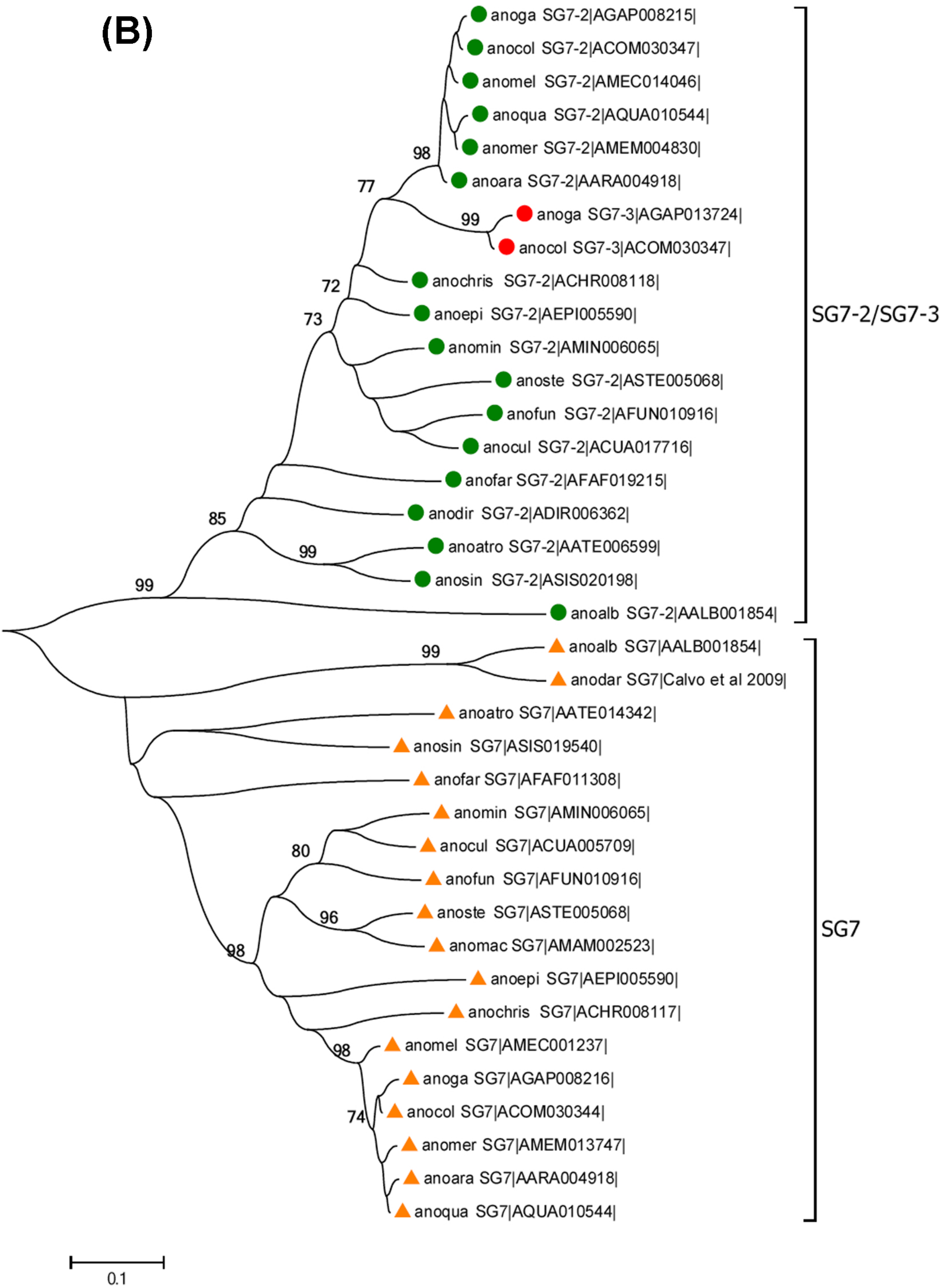
(B)
anoga SG7-2|AGAP008215|
anocol SG7-2|ACOM030347|
anomel SG7-2|AMEC014046|
anoqua SG7-2|AQUA010544|
anomer SG7-2|AMEM004830|
anoara SG7-2|AARA004918|
anoga SG7-3|AGAP013724|
anocol SG7-3|ACOM030347|
anochris SG7-2|ACHR008118|
anoepi SG7-2|AEPI005590|
anomin SG7-2|AMIN006065|
anoste SG7-2|ASTE005068|
anofun SG7-2|AFUN010916|
anocul SG7-2|ACUA017716|
anofar SG7-2|AFAF019215|
anodir SG7-2|ADIR006362|
anoatro SG7-2|AATE006599|
anosin SG7-2|ASIS020198|
anoalb SG7-2|AALB001854|
SG7-2/SG7-3
98
77
99
72
73
85
99
99
anoalb SG7|AALB001854|
anodar SG7|Calvo et al 2009|
anoatro SG7|AATE014342|
anosin SG7|ASIS019540|
anofar SG7|AFAF011308|
anomin SG7|AMIN006065|
anocul SG7|ACUA005709|
anofun SG7|AFUN010916|
anoste SG7|ASTE005068|
anomac SG7|AMAM002523|
anoepi SG7|AEPI005590|
anochris SG7|ACHR008117|
anomel SG7|AMEC001237|
anoga SG7|AGAP008216|
anocol SG7|ACOM030344|
anomer SG7|AMEM013747|
anoara SG7|AARA004918|
anoqua SG7|AQUA010544|
SG7
99
80
96
98
98
74
0.1

(C)

```
30kDa     ----MKFLLLVASVLCLVLIVSARPADDTSDQESSTELSEDAGAEEGAEDAGSDAEADAGAADGEEGATD
gSG7      MHAKPAFVLIALGVICLLQTTPTSASANHVQQ--------------------------------------
gSG7-2    MTIKLCWVFAALGLIWLLQISPCDAGPRHAKQ--------------------------------------
gSG7-3    MSIKLCWVLAALGLICLLQISPSEGTPSHSQQ--------------------------------------
                ::: . .:: *:            .*

30kDa     TESGAEGDDSEMDSAMKEGEEGAGSDDAVSGADDETEESKDDAEEDSEEGGEEGGDGASGGEGGEKESPR
gSG7      ----------------------------------------------------------------------
gSG7-2    ----------------------------------------------------------------------
gSG7-3    ----------------------------------------------------------------------

30kDa     NTYRQVHKLLKKIMKVDTKDKYLKSFVVGRLQERLMNPTIDLVSTIEKYSKI----KECFSSLDKDVSAM
gSG7      --LMKVFRSMVQNFDYTKKPTYLQRA-KYGVQNQLRNPLVQKAGNLPKSAKL---SDGCLKQMVARVTDL
gSG7-2    --LISYFKRMK--LDQTKNRVYQHDV-KNGLRVHLRGPLLQKALCLPKGTKL---SSDCLNRMVDKARQH
gSG7-3    --LLSYFKRMK--LDKTKNRVYLDDV-EYGLRTNLRRPLLQNALFLPKGTKMSVFSSDCLNRMVDKARQH
              . .: :   :.  .:  * .      ::.*  * ::.  : * :*:    . *:. :   .

30kDa     VKE--SEKSYEECSKDKTNTSCGTEGTRELDDGLIEREQELSDCIVDKRDAE
gSG7      EASFYASFSYNCHDHDQYSMECLEAAEPKYLDGLKTLADETAQCLRDQQ---
gSG7-2    ENKFYAQFTYACKTNAEYSAKCLDSGRPVYYHALQKLAKETERCWKL-----
gSG7-3    ENKFYAQFTYASKTNAEYSSDCLDTGRPVYYRDLKKLAKETEQCWKL-----
            .  :. :*    . : . .*   .       *    .*   *
```
