# Supplementary material for: Anopheline salivary protein genes and gene families: an evolutionary overview after the whole genome sequence of sixteen Anopheles species
Source: BMC Genomics. 2017 Feb 13;18:153. doi: 10.1186/s12864-017-3579-8 (PMC5307786; doi:10.1186/s12864-017-3579-8)
Supplement: Additional file 11: — Alignment of mosquito 55.3 kDa/56.5 kDa proteins. Multiple alignment of the 16 full-length anopheline 55.3 kDa family members and the three 56.5 kDa proteins from Cx. pipiens quinquefasciatus, Ae. aegypti and Ae. albopictus. Cysteins are highlighted in red and other fully conserved residues in yellow. Residues conserved in at least seventeen of the nineteen aligned sequences (~90) are highlighted in green. The Highly conserved block of 27 amino acids toward the N-terminus is boxed. Species names are abbreviated with the first three letters of the generic name and the first three-four letters of the specific name. Species names are abbreviated as in Additional file 5 and followed by VectorBase accession numbers (when available). (PDF 121 kb) [file 12864_2017_3579_MOESM11_ESM.pdf]

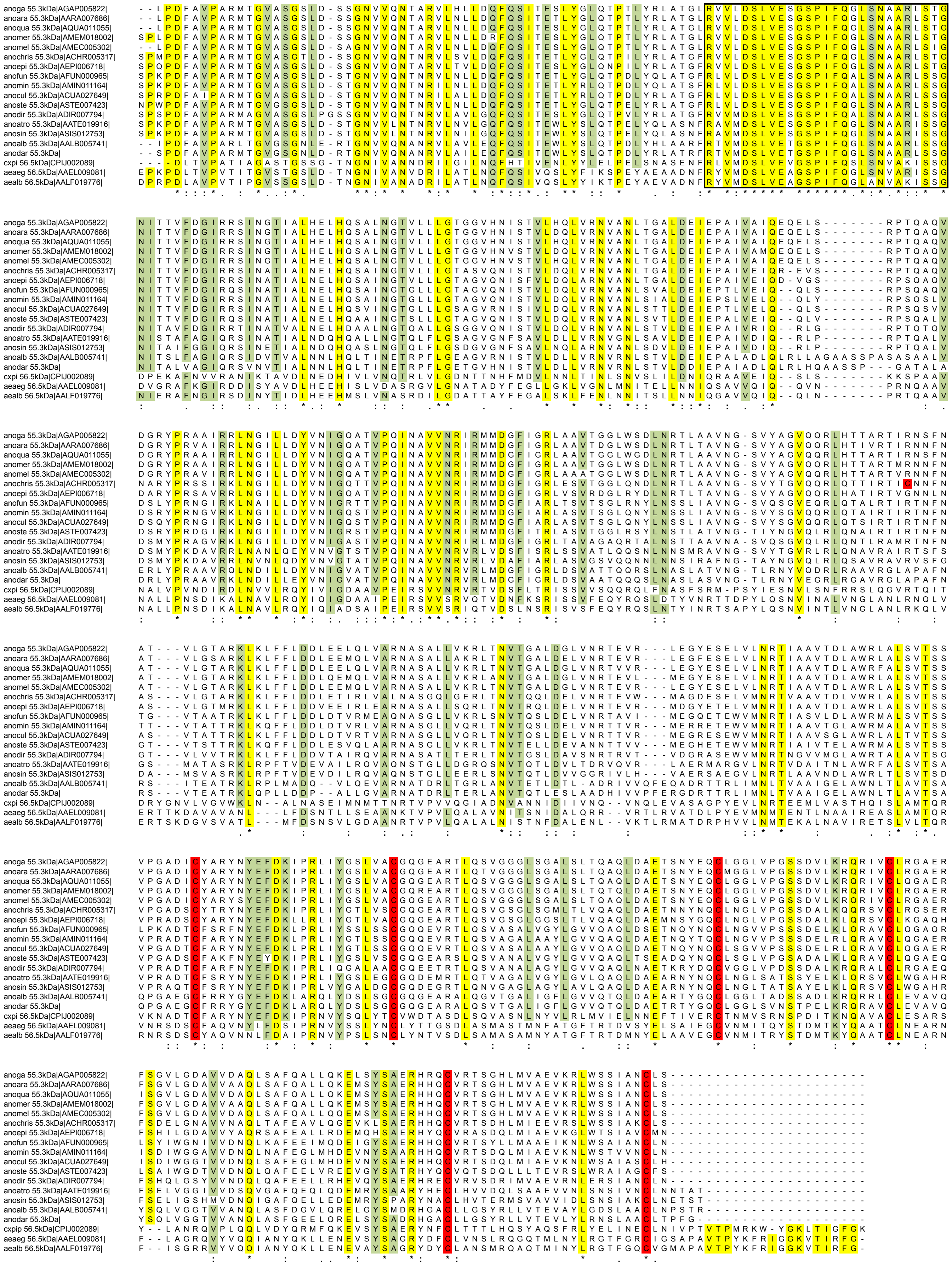

anoga 55.3kDa|AGAP005822|
anoara 55.3kDa|AARA007686|
anoqua 55.3kDa|AQUA011055|
anomer 55.3kDa|AMEM018002|
anomel 55.3kDa|AMEC005302|
anochris 55.3kDa|ACHR005317|
anoepi 55.3kDa|AEPI006718|
anofun 55.3kDa|AFUN000965|
anomin 55.3kDa|AMIN011164|
anocul 55.3kDa|ACUA027649|
anoste 55.3kDa|ASTE007423|
anodir 55.3kDa|ADIR007794|
anoatro 55.3kDa|AATE019916|
anosin 55.3kDa|ASIS012753|
anoalb 55.3kDa|AALB005741|
anodar 55.3kDa|
cxpi 56.5kDa|CPIJ002089|
aeaeg 56.5kDa|AAEL009081|
aealb 56.5kDa|AALF019776|
cxpip 56.5kDa|CPIJ002089|
